# Prognostic enrichment for early-stage Huntington’s disease: An explainable machine learning approach for clinical trial

**DOI:** 10.1016/j.nicl.2024.103650

**Published:** 2024-08-10

**Authors:** Mohsen Ghofrani-Jahromi, Govinda R. Poudel, Adeel Razi, Pubu M. Abeyasinghe, Jane S. Paulsen, Sarah J. Tabrizi, Susmita Saha, Nellie Georgiou-Karistianis

**Affiliations:** a Turner Institute for Brain and Mental Health, Monash University, Clayton VIC3800, Australia; b Mary MacKillop Institute for Health Research, Australian Catholic University, Melbourne VIC3000, Australia; c Department of Neurology, University of Wisconsin-Madison, 1685 Highland Avenue, Madison, WI, USA; d UCL Huntington’s Disease Centre, UCL Queen Square Institute of Neurology, UK Dementia Research Institute, Department of Neurodegenerative Diseases, University College London, London, UK

**Keywords:** Huntington’s Disease, Biomarkers, Neuroimaging, Stratification, Clinical Trials, Machine Learning

## Abstract

**Background:**

In Huntington’s disease clinical trials, recruitment and stratification approaches primarily rely on genetic load, cognitive and motor assessment scores. They focus less on *in vivo* brain imaging markers, which reflect neuropathology well before clinical diagnosis. Machine learning methods offer a degree of sophistication which could significantly improve prognosis and stratification by leveraging multimodal biomarkers from large datasets. Such models specifically tailored to HD gene expansion carriers could further enhance the efficacy of the stratification process.

**Objectives:**

To improve stratification of Huntington’s disease individuals for clinical trials.

**Methods:**

We used data from 451 gene positive individuals with Huntington’s disease (both premanifest and diagnosed) from previously published cohorts (PREDICT, TRACK, TrackON, and IMAGE). We applied whole-brain parcellation to longitudinal brain scans and measured the rate of lateral ventricular enlargement, over 3 years, which was used as the target variable for our prognostic random forest regression models. The models were trained on various combinations of features at baseline, including genetic load, cognitive and motor assessment score biomarkers, as well as brain imaging-derived features. Furthermore, a simplified stratification model was developed to classify individuals into two homogenous groups (low risk and high risk) based on their anticipated rate of ventricular enlargement.

**Results:**

The predictive accuracy of the prognostic models substantially improved by integrating brain imaging features alongside genetic load, cognitive and motor biomarkers: a 24 % reduction in the cross-validated mean absolute error, yielding an error of 530 mm^3^/year. The stratification model had a cross-validated accuracy of 81 % in differentiating between moderate and fast progressors (precision = 83 %, recall = 80 %).

**Conclusions:**

This study validated the effectiveness of machine learning in differentiating between low- and high-risk individuals based on the rate of ventricular enlargement. The models were exclusively trained using features from HD individuals, which offers a more disease-specific, simplified, and accurate approach for prognostic enrichment compared to relying on features extracted from healthy control groups, as done in previous studies. The proposed method has the potential to enhance clinical utility by: i) enabling more targeted recruitment of individuals for clinical trials, ii) improving post-hoc evaluation of individuals, and iii) ultimately leading to better outcomes for individuals through personalized treatment selection.

## Highlights

• A novel machine learning pipeline for predicting ventricular enlargement in Huntington’s Disease.
• Genetic, cognitive, motor, and imaging-derived biomarkers are integrated to achieve more accurate and personalized prognosis.
• Increased homogeneity of recruited participants for clinical trials in early stages of Huntington’s Disease.
• Explainable AI applied for clinical reliability of the proposed stratification model.

## Graphical abstract

**Figure:**
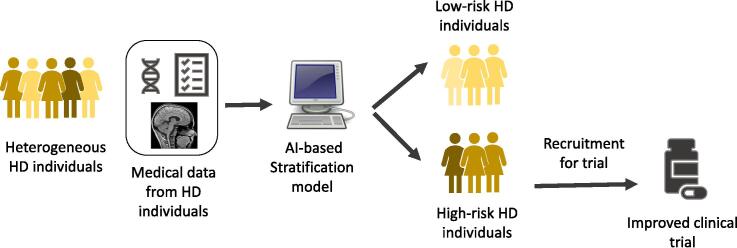

## 1 Introduction

Huntington's disease (HD) is an inherited disorder characterized by a prolonged asymptomatic phase of insidious neuropathological brain tissue damage, followed by episodes of behavioural complications, and ultimately progressive decline in cognitive and motor functions (Ghosh et al., 2017). It is caused by an expansion of the cytosine-adenine-guanine (CAG) trinucleotide repeat in a mutated huntingtin gene (mHTT), which leads to aggregation of toxic proteins in the brain (Haider et al., 2017). HD treatments, to date, are palliative and an effective disease-modifying treatment is yet to be found. The pre-symptomatic (pre-HD) stage of the disease is an important period during which a preventive intervention against brain tissue deterioration could be mostly effective (Kim et al., 2021, Jamwal et al., 2020).

A significant challenge in HD drug trials stems from the heterogeneity among HD gene expansion carriers (HDGECs), highlighting a crucial need for improved stratification to categorize trial participants into more homogenous groups (Paulsen et al., 2019). A solution, advocated by the FDA, is prognostic enrichment (Fda, 2019, Maheux et al., 2023). which aims to strategically identify and include individuals with higher risk of experiencing severe disease progression. This results in more consistent responses to experimental treatments thereby facilitating the ability to detect treatment effects (Oxtoby et al., 2022).

Currently, stratification of individuals in HD clinical trials mostly relies on genetic load, calculated by CAG and age product known as the CAP score (Warner et al., 2022), as well as cognitive, motor, and functional assessment scores that are quantitatively encapsulated in the composite Unified Huntington’s Disease Rating Scale (cUHDRS) (Estevez-Fraga et al., 2021). Another weighted combination of these biomarkers is proposed in the normalized prognostic index (PIN) (Long et al., 2017), specifically tailored to enrich a recruited population by providing a predictive assessment of future motor diagnosis risk. Although the number of CAG repeats is a significant determinant of HD onset and progression rate, the specific effect differs broadly among individuals (Reilmann et al., 2019). In recent years, advanced assays for accurately measuring biofluids in the blood and cerebrospinal fluid have promised objective biomarkers (McColgan et al., 2022), and genetic modifiers of CAG repeats are continually investigated (Lee et al., 2022, Tabrizi et al., 2022). Nevertheless, *in vivo* brain imaging remains a reliable source of information that can reflect the severity of the latent neuropathologic state of individuals with HD (Kinnunen et al., 2021). PREDICT-HD (Paulsen et al., 2014, Ciarochi et al., 2016), TRACK-HD (Tabrizi et al., 2013), and IMAGE-HD (Poudel et al., 2015, Poudel et al., 2019) are longitudinal observational imaging studies that aimed to identify sensitive biomarkers (Paulsen et al., 2008), predict onset (Langbehn et al., 2010), profile the natural history (Abeyasinghe et al., 2021), and improve endpoints for HD clinical trials (Paulsen et al., 2010). These studies have generated a wealth of information, now being leveraged for other investigations. For example, built upon these studies and others, the recently developed data-driven HD Integrated Staging System (HD-ISS) (Tabrizi et al., 2022) has recognized the volumes of caudate and putamen as key biomarkers of pathogenesis. This has opened up the possibility of recruiting HDGECs, both pre-HD and symptomatic HD (symp-HD), for preventive trials by using HD-ISS stages 0, 1, and 2, that precede traditional Shoulson-Fahn stages (now incorporated in HD-ISS stage 3) (Shoulson and Fahn, 1979). However, HD-ISS serves as a first-stage classifier and further granular differentiation of HDGECs is required.

While the consideration of caudal and putaminal volumes in HD-ISS stage 1 is a major leap, brain deterioration in HD is not limited to the striatum, and other subcortical structures and cortical regions are also impacted during the disease course (Johnson et al., 2021, Tan et al., 2021, Van Den Bogaard et al., 2011). Generally, atrophy in deep and cortical regions is accompanied by a quadratic-like (Mofrad et al., 2021, Bethlehem et al., 2022) enlargement of the lateral ventricles, which are filled with cerebrospinal fluid (CSF) in order to compensate for tissue loss and preserve total intracranial volume (ICV) (Nestor et al., 2008, Mak et al., 2017). As a result, ventricular volume has been commonly used as a surrogate measure of brain atrophy in HD clinical trials (Tabrizi et al., 2019, UniQure Biopharma, 2019, Novartis-Pharmaceuticals, 2021) and other brain disorders (Petracca et al., 2121, Apostolova et al., 2013). One study found that incorporating the volume of lateral ventricles, as one of the inputs of a classification model that aimed for HD stage identification, significantly improved the discriminative power, as compared to the same model being trained solely with features derived from the striatum (Kohli et al., 2022, Kohli et al., 2023).

During the past decade, alongside the abundance of multi-modal data in neurodegenerative disorders, there has been a proliferation of artificial intelligence (AI) models for clinical support in diagnosis, prognosis, and treatment management (Bron et al., 2022, Kopf and Claassen, 2021, Mestre, 2022, Amirmoezzi et al., 2019). Machine learning models have proved the feasibility of: predicting the future motor state of pre-HD individuals from baseline scans (Castro et al., 2017); introducing new disease states within its course (Mohan et al., 2022); and improvements for phase II or III clinical trials (Koval et al., 2022). Moreover, predictive models of neurodegenerative conditions have become more clinically viable by incorporating Explainable AI, which is crucial for providing transparency and understanding of the underlying processes within such models, thereby increasing trust in their predictions (Schulz et al., 2020, de Jong et al., 2021, Antoniadi et al., 2022, Junaid et al., 2023, Rasheed et al., 2022).

This study presents a machine learning pipeline based on random forests (Strobl et al., 2009, Ghazaleh et al., 2021) to predict the enlargement rate of lateral ventricles within a 3-year time window. We took advantage of the nonlinear growth of lateral ventricles to identify fast-progressor HDGECs, especially in HD-ISS stages 1, and 2. For this purpose, we conducted a search for a novel scale aimed at stratifying moderate and fast progressor groups into more granular levels of individual risk. We hypothesized that random forests could find homogenous subspaces spanned by features from HD genetic load, cognitive, motor, and volumetric imaging biomarkers to achieve more accurate predictions of brain atrophy. The clinical utility of our proposed approach is to provide: 1) an HD-specific and early-stage-targeted prognostic enrichment and subsequent stratification tool for enhancing sample homogeneity based on predicted brain atrophy, facilitating the selection of participants for future HD clinical trials, 2) a patient-specific prognostic model enabling individualized post-hoc assessment of treatment effects, and 3) inter-variable relationships among biomarkers of HD progression.

## 2 Materials and methods

### 2.1 Data preparation

We used data from previously published cohorts: PREDICT (Paulsen et al., 2013), TRACK (Tabrizi et al., 2009), TrackON (Klöppel et al., 2015), and IMAGE (Jfd et al., 2013). The datasets involved repeated visits, with structural MRI scans being acquired along with genetic, cognitive, motor and functional assessments. Table 1 presents a summary of the baseline data regarding demographic information, CAG repeats, cognitive and motor assessments, as well as the length of study engagement (years), separated by cohorts. We limited our analyses to images obtained exclusively from 3Tesla scanners. Pre-processing and quality control were conducted similarly to our earlier study (Abeyasinghe et al., 2021), in which FreeSurfer (Reuter et al., 2010) was employed for subcortical segmentation, cortical parcellation and extraction of imaging-derived features. Volumes of deep and cortical regions were divided by ICV, while cortical thickness measurements were used without normalization as suggested in the literature (Westman et al., 2013). Longitudinal change of hemispheric differences in HD-related atrophy is demonstrated to be limited (Wijeratne et al., 2020), therefore, within our analyses we merged the volumes of subcortical structures from both hemispheres.

**Table 1:** Demographic characteristics of Huntington’s Disease individuals at baseline.

	TRACK		PREDICT		IMAGE		Aggregated Data	
	**pre-HD**	**symp-HD**	**pre-HD**	**symp-HD**	**pre-HD**	**symp-HD**	**pre-HD**	**symp-HD**
N	156	111	106	12	35	31	297	154
%Female	55 %	55 %	59 %	75 %	60 %	35 %	58 %	53 %
Age	40.2 (9.0)	49.6 (9.4)	42.5 (12.4)	52.0 (11.7)	41.8 (9.0)	52.6 (9.4)	41.2 (10.5)	50.4 (9.6)
CAG	43.2 (2.4)	43.4 (2.8)	42.2 (2.5)	42.4 (2.8)	42.2 (1.9)	42.9 (2.1)	42.7 (2.5)	43.2 (2.7)
TMS	3.1 (2.8)	23.7 (10.8)	5.3 (5.2)	25.7 (14.1)	0.8 (1.1)	15.2 (6.8)	3.6 (4.0)	22.9 (11.0)
SDMT	51.6 (10)	34.0 (10.3)	52.1 (10.8)	39.2 (12.3)	52.7 (9.2)	42.1 (11.0)	51.9 (10.3)	35.6 (10.9)
SWR	100 (16.8)	77.9 (19.5)	99.2 (15.8)	77.7 (29)	103 (17.2)	93.9 (17.9)	100 (16.5)	80 (20.8)
LVER	680 (6 1 7)	2166 (1521)	707 (8 4 7)	2041 (1284)	532 (6 4 1)	2100 (1296)	673 (7 1 0)	2143 (1453)
Engagement	3.78 (1.85)		2.76 (1.45)		2.47 (0.47)			

Mean (SD) for quantitative variables and count or percentage for categorical variables. CAG: cytosine-adenine-guanine repeats, TMS: Total Motor Score, SDMT: Symbol Digit Modalities Test, SWR: Stroop Word Reading test, LVER: Lateral Ventricular Enlargement Rate (mm^3^/year). Engagement refers to the duration, in years, between the initial and final MRI acquisition.

### 2.2 Input feature domains

**Fig.1:**
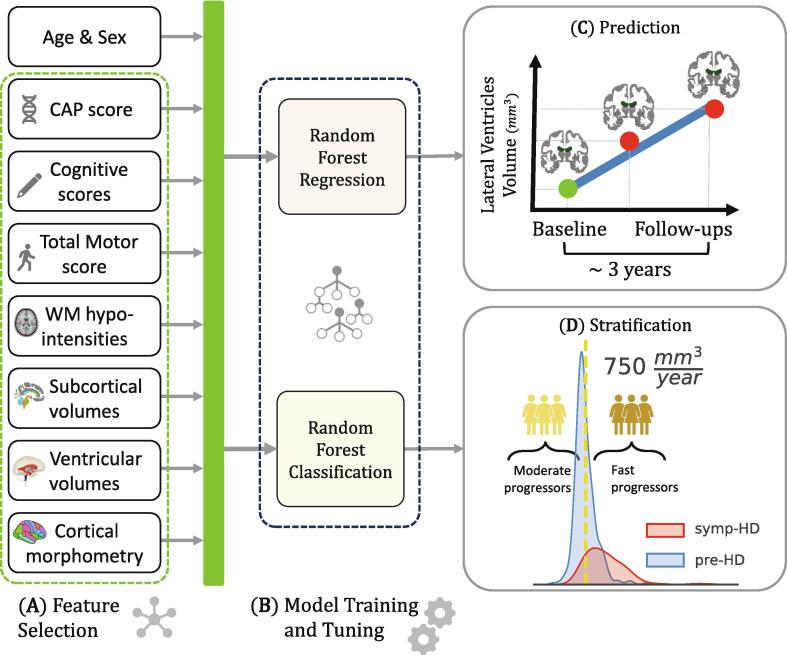
**An overview of the pipeline for training the machine learning models for prognosis and stratification. (A)** Within the sequential feature selection, biomarkers from different domains were incrementally added to the input feature set and fed into the models for training. **(B)** The random forest models were trained and then evaluated on an independent test set for each subset of selected features. **(C)** For each individual, the rate of forthcoming lateral ventricular enlargement was calculated using linear regression analysis and selected as the ground truth for the prognostic random forest regression model. **(D)** To stratify the population into two homogenous groups, a classification task was defined as forecasting whether an individual’s prospective rate of ventricular enlargement would be below or over a threshold of 750 mm^3^/year, which was set to be close to the median value to avoid class imbalance.

Initially, the method of Minimum Redundancy Maximum Relevance (Peng et al., 2005) was used to identify key predictor domains and remove those features that were the least informative in association with ventricular volume. At this step, cortical surface area and curvature were excluded from the feature set. The remaining features, categorized into seven domains, are depicted in Fig. 1A. The features in each domain were not combined into a single parameter. Instead, to assess the impact of each domain on model performance, we sequentially incorporated them into the complete input feature set and recorded performance metrics. These feature domains comprised the CAP score, calculated as [Age × (CAG-35.5)] (Mason et al., 2018), UHDRS-TMS, cognitive biomarkers including symbol digit modalities test (SDMT) and Stroop Word Reading Test (SWR) (Poudel et al., 2014, Andre et al., 2014), together with 162 imaging derived features grouped into subcortical, cortical, and ventricular volumes (see Supplementary Table S1 for details). The hallmark structures affected in HD (Tan et al., 2021), including caudate, putamen, pallidum, thalamus, hippocampus, amygdala, accumbens, and corpus callosum were grouped together as the subcortical domain. Features related to the ventricular system involved the volumes of the lateral, inferior lateral, the 3rd and the 4th ventricles, and CSF. Our analyses showed that the choroid plexus was significantly larger in the later HD-ISS stages (see section 3 in the supplementary materials), so we included this feature in this group as well. We categorized both the volumes and thicknesses of 34 cortical regions as cortical morphometry features, each of which present bilaterally, based on the Desikan-Killiany atlas (Desikan et al., 2006). The volume of white matter hypo-intensities on T1-weighted images, which has been reported to be strongly correlated with T2-weighted hyper-intensities and to be also associated with age and pathology (Wei et al., 2019), was included as a feature domain in our analyses. Supplementary Fig. S1 illustrates how this feature is distributed across different groups and cohorts in this study.

### 2.3 Prognostic model of lateral ventricular enlargement

The forthcoming lateral ventricular enlargement rate (LVER) over time was considered as an indicator of brain tissue loss (Lundervold et al., 2019). It was calculated using linear regression analysis for each individual separately, as schematically shown in Fig. 1C. Subsequently, LVER served as the dependent variable for training random forest regression models, using scikit-learn, a python library for machine learning (Pedregosa et al., 2011). Clinical trials are typically conducted over periods not exceeding 4 years (Estevez-Fraga et al., 2024, Kinnunen et al., 2021). For those individuals in PREDICT and TRACK, who participated in the studies for periods longer than 4 years, the longitudinal data was divided into two shorter segments, each segment having its own baseline features and corresponding calculated LVER value. However, it is noteworthy that this decision was made to obtain more consistent LVER calculations; our post-hoc analyses revealed that separation into segments did not significantly impact the model accuracy. For more details, see Section 17 in the supplementary materials. Extra care was taken to ensure exclusive allocation of these segments to either training or test sets. This technique yielded 537 input–output pairs, 80 % allocated for training, 10 % for validation, and 10 % for test.

To examine the prognostic value of each feature domain, we employed a wrapper-based sequential forward feature selection approach (Ruiz et al., 2006), where each domain was incrementally added to the input feature subset of the training dataset. The model was trained and tuned at each step, and the effect on the reduction of the prediction error in the prognostic model was recorded for both a 10-fold cross-validation setting and an independent unseen test set. Seven prognostic models were trained, each with its hyperparameters being tuned. Age and sex were included in the input feature set for all models to account for their potential confounding effect; similarly, ICV was also included in models V, VI, and VII which had imaging features.

Both feature selection and hyperparameter tuning processes were carried out using only the training dataset, with the test set remaining untouched. The number of constituent decision tree estimators in the random forest was 300 and the tuned hyperparameters included the maximum number of leaf nodes, minimum number of samples in each leaf, and the maximum number of samples and features being used for training.

### 2.4 Stratification model for moderate and fast progression

To evaluate the predictive power of the proposed pipeline, we assessed its capability to stratify the population in the pooled dataset into two groups: moderate and fast progressors, based on their anticipated brain atrophy. The distribution of ground truth LVERs across the pooled dataset is illustrated in Fig. 1D, with a detailed depiction of the distribution over HD-ISS stages provided in Supplementary Fig. S2. We selected the median LVER (750 mm^3^/year) as the reference threshold for classification. These stratification models were trained for prognostic enrichment in clinical trials using baseline features used for the prognostic models, but with the aim of identifying individuals who are more likely to exhibit fast disease progression and screening out those projected to experience moderate progression. The assumption here is that the HD continuum is sufficiently represented by the 537 baseline samples in the pooled dataset and that a random forest classifier could partition the space spanned by the input features into homogenous subspaces not relying on the individuals’ motor diagnosis, but rather based on the expected rate of pathologic neurodegeneration.

We used Shapley additive explanations (SHAP) (Lundberg and Lee, 2017) to reveal the basis for the classification outcomes and to demonstrate the contribution of input features, their importance, and interactions.

## 3 Results

### 3.1 Evaluation of the prognostic model

**Fig.2:**
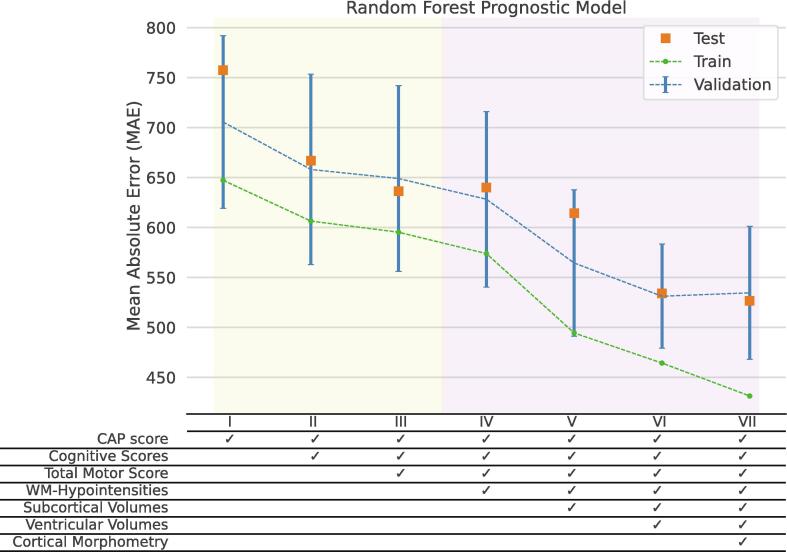
**Sequential integration of biomarker domains into the feature set.** By successively integrating each domain of biomarkers in the input features set, the error and the uncertainty of the prognostic models dropped. Mean absolute error is computed by taking average of deviations between true and predicted values of lateral ventricular enlargement rate in the train, validation, and test set. The test set comprised 53 samples, unseen during training, and was used to assess the generalizability of the model.

The prognostic model was repeatedly trained and evaluated while the input feature set sequentially incorporated the seven defined domains. Fig. 2 illustrates how the inclusion of each domain reduced the mean absolute error (MAE) in the predictions. The first three models, highlighted by yellow, were fed with features conventionally used for recruitment in clinical trials (CAP, SDMT, SWR, and TMS). By incrementally appending the imaging-derived features to the input set, at each step, a sharp decline was seen in the prediction error. For instance, by including the volume of white matter hypo-intensities, the cross-validated prediction error reduced by 3 %. The reduction was 24.6 % when comparing Model VI to Model I (refer to Supplementary Table S2). The error bars represent the standard deviation of MAE in a 10-fold cross validation setting. Alongside the improvement in predictive power, the uncertainty of the models, measured by coefficients of variation in MAE, also dropped: 0.13 for model I but 0.09 for model VI. This highlights how objectively measured imaging biomarkers compensated for the interrater variability and subjectivity of cognitive and motor assessments (Scheid et al., 2022). A similar trend of error reduction was seen in the unseen test set, shown in orange. It is worth noting that the hyperparameters were tuned solely to prevent overfitting to the training data, a common issue when random forests are used for regression tasks. This tuning procedure did not significantly affect evaluation metrics. A regularized linear regression model with considerably fewer hyperparameters showed a similar trend of MAE reductions, but with a smaller difference between train and test metrics (Supplementary Fig. S3).

### 3.2 Performance of the stratification model

Stratification models were trained with the aim of segregating the pooled dataset into two groups of moderate and fast progressors. A classifier with input features and hyperparameters similar to prognostic model VI, could achieve the maximum accuracy of 0.77 on the independent test set and 0.81 within cross validation (precision = 0.83, recall = 0.80). The choice of the stratification threshold can be customized based on the specific clinical requirements. Supplementary Fig. S6 illustrates the relationships between the selected threshold and specificity, sensitivity, and accuracy which could be useful in fine-tuning the model's performance and selecting an optimal threshold that balances the specificity-sensitivity trade-off according to a study's goals. This allows selection of the threshold based on whether the focus in enrichment is to screen out individuals with moderate progression or prioritize the identification of fast progressors for inclusion.

**Fig.3:**
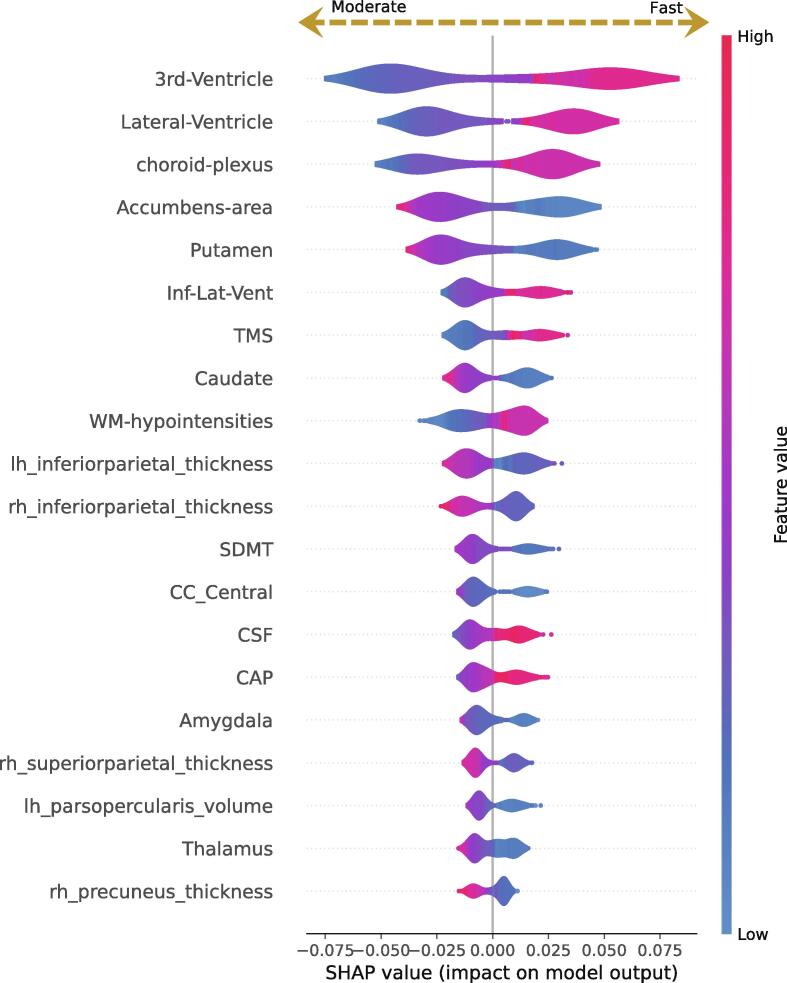
**Shapley additive explanations computed for the stratification model.** Features are ranked based on their contribution to class predictions made by the trained model. Each feature is represented by a spectrum ranging from blue to red, with blue tones indicating lower feature values and red tones indicating higher values. The left and right ends of each spectrum correspond to moderate and fast lateral ventricular enlargement. The results demonstrate how imaging biomarkers can enhance the predictive power of conventional clinical and cognitive scores, allowing for more accurate homogenization and stratification of Huntington’s disease cohorts. (Inf-Lat-Vent: Inferior Lateral Ventricle, CC_central: central subdivision of corpus callosum).

Fig. 3 displays the SHAP values of the stratification counterpart of prognostic model VII, where the features are ranked according to their importance. The left and right ends of the spectrum represent moderate and fast LVERs, respectively. Red tones indicate higher values of each specific feature, while blue tones indicate lower values. The results validate the model's ability to learn and align with the clinical research findings regarding the pathological HD processes, including ventricular enlargement, subcortical atrophy, cortical thinning, and alterations in cognitive and motor scores. For example, higher TMS values associate with faster rates of ventricular enlargement; however, as compared to the red tones, blue tones have an extensively narrower distribution. This is due to the fact that TMS values for most individuals in the early stages of disease are near zero (Table 1), resulting in a skewness in TMS distribution known as the floor effect (Abreu et al., 2021). In the proposed pipeline, this issue is compensated by the contribution of features in rows above TMS, i.e., the ventricular, putaminal, and accumbal volumes (Fig. 3), which exhibit more symmetrical distributions compared to TMS and SDMT.

### 3.3 Prognostic enrichment based on lateral ventricular enlargement rate

**Fig.4:**
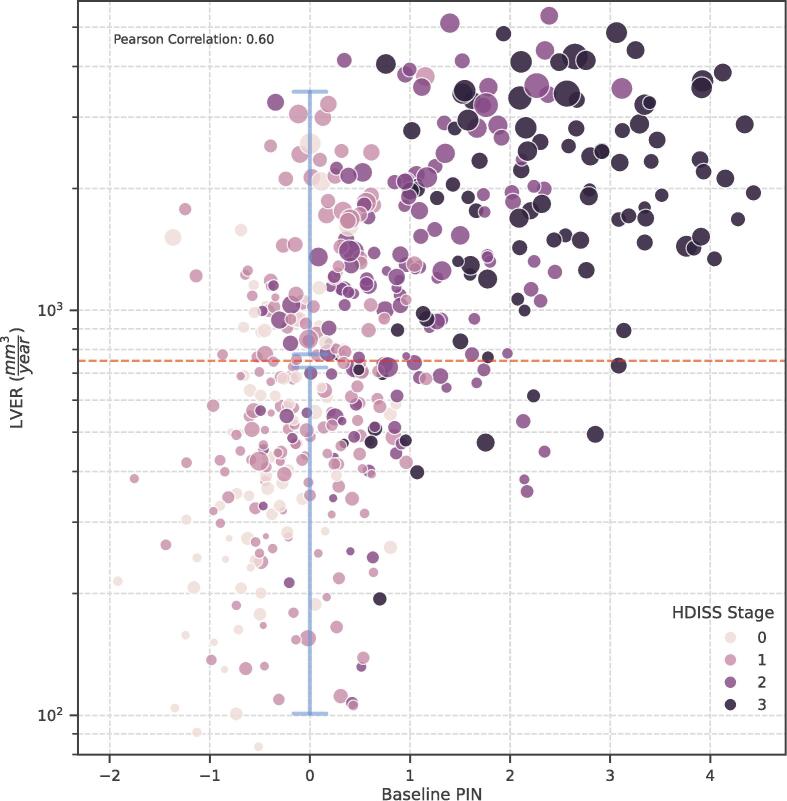
**Association between baseline Normalized Prognostic Index (PIN) and the forthcoming lateral ventricular enlargement rates (LVER).** While the values of LVER are associated with the PIN scores, there exists extensive variability in LVER values across any specific range of PIN scores. For instance, PIN scores near zero encompass a wide range of 100 to 3000 mm^3^/year for forthcoming LVER. In clinical trials, where participants are primarily stratified based on their PIN scores, recruitment can be further enriched by employing the proposed models to select individuals with narrower ranges of predicted LVER, thereby achieving a more homogeneous population. The size of the circles corresponds to the volume of lateral ventricles at baseline, normalized by intracranial volume.

Fig. 4 illustrates the relationship between the baseline PIN score calculated using the formula given by Long *et al*. (Long et al., 2017) and the ground truth values of LVER. There exists extensive variability in LVER values across any specific range of PIN scores. This is due to the fact that the enlargement of the ventricular volume generally follows a quadratic-like pattern compared to caudate or putamen that atrophy linearly (Hobbs et al., 2010). For details, see Section 16 in the supplementary materials. In a trial that targets a specific range of PIN scores, the characteristics of recruited participants may become more homogenous by narrowing down participant selection based on the anticipated values of LVER. This can be achieved by employing the proposed pipeline which enables training and validating both the prognostic and the stratification models using any combination of the input feature domains. In a scenario, where imaging features were fully absent in the input feature set, the stratification model achieved a cross-validated accuracy of 0.72. Conversely, feeding the model exclusively with the imaging-derived features resulted in a higher cross-validated accuracy of 0.81. Refer to Supplementary Table S2 and Fig. S10 for details of the model evaluation.

**Fig.5:**
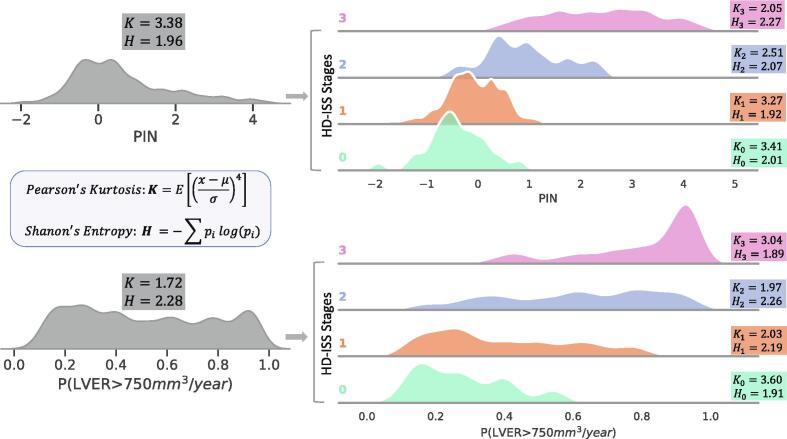
**Comparative Analysis of Prognostic Metrics within HD-ISS Stages.** Here, in a leave-one-out setting, for each individual, the prognostic model is trained using imaging data from other individuals and then employed for prediction. Baseline samples are stratified by HD-ISS stages. In stages 1 and 2, the proposed prognostic metric, P(LVER>750 mm^3^/year), exhibits a broader and flatter distribution than PIN (lower kurtosis). Equivalently, higher entropy indicates more diverse and less concentrated values, suggesting the applicability of P(LVER>750 mm^3^/year) for prognostic enrichment in stages 1 and 2. The proposed prognostic metric does not provide value over PIN for stage 0 and can be marginally beneficial in stage 3 for screening out slow-progressors.

A probabilistic perspective of the stratification model can be attained by quantifying its certainty in determining whether an individual with HD has surpassed the LVER threshold of 750 mm^3^/year. This is determined by the consensus among decision trees within a random forest. Higher certainties signify a more robust consensus among the trees, indicating a greater inclination towards classifying an individual as surpassing the threshold. Fig. 5 compares the model’s certainty, denoted as P(LVER>750 mm^3^/year), with the PIN score. Pearson’s kurtosis (K) and Shannon’s entropy (H) are used to quantify the distribution characteristics. Lower kurtosis and higher entropy correspond to a greater level of distinction among the samples and less concentration around the mean. While the PIN score demonstrates a high level of distinction among individuals in HD-ISS stage 3, characterized by lower kurtosis and higher entropy, it lacks the ability to strongly discriminate between individuals in earlier stages. Conversely, the proposed probabilistic metric offers extensive distinction among individuals in HD-ISS stages 2 or 1, albeit at the expense of reduced discriminatory power in HD-ISS stage 3.

### 3.4 Associations between cortical degeneration and ventricular enlargement

A comparison between prognostic models VI and VII suggests that incorporating features related to cortical morphometry may not significantly enhance the predictive power of the models. Nevertheless, model explanations demonstrated that several cortical morphometry features are informative for discriminating between moderate and fast disease progression.

In two separate analyses, we trained the models exclusively based on cortical measurements. Once fed with cortical thickness features, the cross-validated accuracy was 0.73 ± 0.04 (precision = 0.74, recall = 0.77), whereas it was 0.68 ± 0.07 (precision = 0.71, recall = 0.69) when trained using cortical volumes.

**Fig.6:**
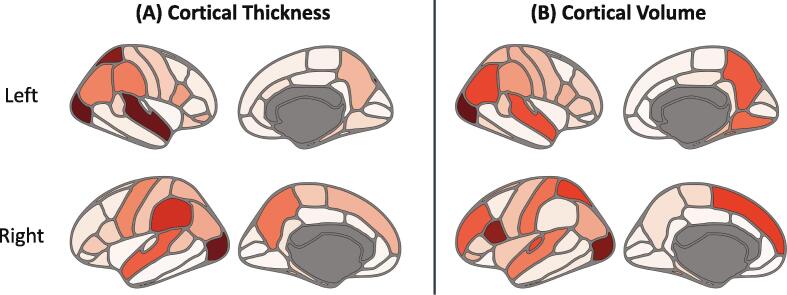
**Cortical regions predictive of moderate and fast progression.** Darker tones correspond to **(A)** cortical thicknesses and **(B)** cortical volumes of regions that had higher impact in predicting fast ventricular enlargement. These regions may potentially serve as monitoring biomarkers to assess treatment response in trials that aim to halt cortical atrophy.

The thicknesses of the lateral occipital, superior temporal and superior parietal and the volumes of lateral occipital, the left pars opercularis and superior parietal cortices were the most important cortical morphometry features, consistent with previous research (Johnson et al., 2021). Degenerated cortical regions ranked by their contributions to model decisions based on SHAP are shown in Fig. 6. Regions depicted in darker tones associate with higher predictive power of the forthcoming atrophy rate and could potentially serve as monitoring biomarkers (FDA-NIH Biomarker Working Group, 2016) within trials similar to PRECREST (Rosas et al., 2014) that aim to slow cortical thinning.

## 4 Discussion

We developed a novel machine learning pipeline fed with multiple combinations of baseline HD biomarkers. We trained and validated regression and classification random forests for prognosis and stratification, respectively. Leveraging longitudinal brain scans alongside conventional HD biomarkers from PREDICT, TRACK, TRACKON and IMAGE, we focused on the accelerated enlargement of lateral ventricles over time as a proxy measure of brain atrophy. We systematically incorporated seven different domains of biomarkers into the input feature set, one at a time. This sequential integration allowed us to evaluate and rank the contribution of each domain to the improvement of predictive accuracy of the models. Particularly, the inclusion of imaging-derived features resulted in significant reductions in both errors and variances of predictions.

We adhered to the latest guidelines that recommend to view the disease course in neurodegenerative disorders as a continuum (JNPD Research and Innovation, 2019), and utilized random forests to classify participants into homogeneous groups of moderate and fast progressors based on their forthcoming brain tissue loss rather than their clinical motor diagnosis. This approach allowed the trained models to extrapolate HD stages without relying on data from neurologically normal individuals, which may introduce additional factors in the model that are related to neurodevelopmental aspects of HD and thus not specific to its neurodegenerative process (Van Der Plas et al., 2020, Kubera et al., 2018). Müller and colleagues proposed a disease progression score derived from a multiplicative and divisive relationship among the volumes of the parietal lobe, striatum, and lateral ventricles (Müller et al., 2018). The proposed model in this study also seeks a nonlinear relationship, however among all input features and LVER to identify HD individuals who are more likely to exhibit considerable ventricular expansion in future. What distinguishes the present study from similar previous data-driven works is that the proposed model is trained exclusively with features from HDGECs, without incorporating data from healthy controls. This choice is grounded in the clinical trial context, where non-gene carriers are not part of the study population and their inclusion in computational modelling schemes does not necessarily guarantee higher accuracy when the goal is segregating HDGECs into homogeneous subgroups. This is different from the common event-based approaches used in modelling the progression of neurodegenerative disorders, including HD (Müller et al., 2018, Wijeratne et al., 2021, Castro et al., 2022, Wijeratne et al., 2023), which is to view the disease course as consisting of two major distinct states: an initial state, where HDGECs are assumed to exhibit characteristic biomarkers similar to healthy individuals, and a subsequent state within which biomarker trajectories in HDGECs are expected to deviate from healthy individuals. The results of HD-YAS study (Scahill et al., 2020, Scahill et al., 2022) suggest that the former assumption holds true for cognitive and motor assessments. However, this may not be the case for cortical measurements, which have been presumed to be affected by the neurodevelopmental component in HD (Tan et al., 2021). An example is presented in Supplementary Fig. S7, where insular thickness was found to be significantly greater in pre-HD group not only compared to those with symptomatic HD, but also to healthy controls. Therefore, in order to enable a machine learning model to capture a holistic view of the dynamics of cortical changes throughout the HD course, it is essential to provide it with data that comprise a broader age range than the aggregated datasets used in this study and also more complicated features extracted from the cortex (Stoebner et al., 2023, Shishegar et al., 2021).

Improving the homogeneity of trial populations is crucial for obtaining reliable results in phase II or III clinical trials, which can be achieved by recruiting participants with less dissimilar prognosed disease progression (Maheux et al., 2023, Oxtoby et al., 2022). We selected random forests as our modelling approach, since their intrinsic design involves recursively partitioning a high-dimensional feature space by minimizing an impurity function in each of the nodes, ultimately resulting in input samples being clustered in optimized subdivisions with similar target values (Strobl et al., 2009), reminiscent of the goal of stratification in clinical applications. We took advantage of the non-parametric architecture of random forests to predict short-term brain atrophy in contrast to previous literature that used parametric models to fit predefined long-term trajectories to the biomarkers in individuals (Koval et al., 2022).

It is likely that while an individual in HD-ISS stage 1 does not exhibit noticeable motor or cognitive problem, their prognosed brain atrophy is higher than others at the same stage. On the other hand, a common practice in interventional clinical trials is to select participants from previously available cohorts, such as Enroll-HD (Long et al., 2023), where imaging data may be unavailable. We ranked the importance of conventional motor and cognitive biomarkers of HD progression with respect to their contributions to the decisions made by the stratification model. Our analysis revealed that the PIN had a higher importance than the CAP, cUHDRS, and their individual components (Supplementary Fig. S9). These findings are consistent with previous studies that reported on the predictive value of the PIN score (Langbehn et al., 2022). Given that PIN has also shown strong association with neurofilament light in HD (Parkin et al., 2022), we suggest that our proposed metric for prognostic enrichment could serve as a complementary tool along with the HD-ISS, after recruited participants are PIN-stratified. In such an arrangement, by leveraging information from imaging-derived features after MRI acquisition, the proposed models can further differentiate between individuals whose PIN scores suggest relatively similar prognosis (Fig.4, Fig.5). Moreover, the probability of anticipated LVER surpassing a certain threshold can be considered as a biomarker of HD progression, with higher probabilities highlighting more severe conditions. Such metrics that incorporate imaging information offer greater robustness, compared to composite scores that are based on cognitive and/or motor assessments that may be susceptible to fluctuations due to practice effect, rater subjectivity, or variability (Supplementary Fig. S11).

Inter-variable relationships among striatal and non-striatal degeneration, cortical thinning, and non-imaging biomarkers based on the value of each feature are presented by SHAP in Fig. 3. In an investigation of the spatiotemporal dynamics of cortical atrophy, Johnson et al. found that occipital and parietal regions undergo significantly higher rates of disease-related degeneration compared to frontal and temporal regions (Johnson et al., 2021). This is consistent with our findings, which indicated that parietal and occipital regions are more predictive of ventricular enlargement rates (Fig. 6).

A published post-hoc analysis of the participants in the GENERATION-HD1 tominersen study revealed that they were a decade older with respect to their brain age (Hawellek et al., 2022). Furthermore, the volumes of the lateral ventricles and choroid plexus have shown to serve as one of the dominant aspects of brain aging (Bethlehem et al., 2022, Smith et al., 2020). Our study confirms the implication of ventricular system as an informative biomarker of accelerated brain atrophy in HD progression. Accordingly, a surrogate endpoint for experimental therapeutics could be the inhibition of pathologic ventricular enlargement, particularly in earlier disease stages. Nevertheless, the applicability of ventricular volume as a measure of trial efficacy is constrained by intervention-induced brain inflammation (Tabrizi et al., 2019). One limitation of using the proposed prognostic model for evaluating treatment effects is that ventricular enlargement may be confounded by processes other than atrophy when the intervention involves intrathecal administration (McColgan et al., 2023). Further investigation of the proposed model with real-world data from clinical trials is essential. In particular, pre- and post-intervention LVER estimations should be compared, with contributions from each imaging-derived feature analysed using SHAP waterfall plots (see supplementary Figures S12 and S13). This potential functionality could facilitate post-hoc trial analysis by enabling a simultaneous comparison of changes in imaging-derived features alongside their corresponding SHAP values in model explanations. Utilizing these subject-specific estimations may also help to minimize, if not eliminate, the requirement for a large number of placebo-receiving participants, addressing the longstanding ethical issue of placebo arms within clinical trials and potentially improving success rates (Largent et al., 2022).

Ventricular expansion due to normal aging is inevitable and may confound the estimations, thereby limiting the applicability of the proposed models. On the other hand, it has been reported that conventional approaches to confound removal may lead to unintended leakage of data resulting in unrealistic improved performance and hindered reproducibility (Hamdan et al., 2022). In order to avoid this issue while accounting for normal aging, we trained a separate prognostic model using imaging domain features from healthy control individuals to decompose the effects of normal and pathogenic neurodegeneration and obtain a “lower bound” for the anticipated ventricular expansion (Schwarz, 2021), which could potentially be used to evaluate if a clinical trial has been successful in slowing brain atrophy (Supplementary Fig. S8).

The training dataset was not adjusted beyond the quality check of the automated brain segmentation. Even instances with negative ventricular change over time were included in the training set to ensure model generalizability. Multi-site participation is typically required for HD clinical trials to achieve sufficient statistical power. Volumetric MRI has shown to be less susceptible to multisite discrepancies than diffusion and functional MRI (Gregory et al., 2020). We also conducted out-of-sample testing for all configurations of using dissimilar cohorts for training and testing and found no significant bias towards a specific cohort (Supplementary Table S8). The use of random forests, which are composed of a large number of decision tree estimators ensembled together, makes the stratification model robust to measurement noise. However, for such AI-based systems to be used in clinical research, it is crucial that all image segmentations be thoroughly reviewed by domain experts to ensure clinical reliability. This is especially important as these systems are typically trained and tuned by non-clinicians.

Another limitation of this study is the absence of biofluid biomarkers in the dataset, which could potentially enhance the accuracy of the prognostic model and increase the sensitivity of the stratification model for identifying pre-HD fast progressors. Furthermore, it is worth exploring whether the inclusion of features related to diffusion or resting-state functional MRI (Polosecki et al., 2020) could improve the models' performance. Another limitation is that the calculation of ground truth values required at least two longitudinal brain scans, which led to the exclusion of several individuals from analysis. However, more advanced techniques, such as generative models, can incorporate data from subjects with only one brain scan as well. Additionally, recent studies have reported that deep learning-based brain segmentation tools (Kemenczky et al., 2022) may be less susceptible to head movement artifacts than traditional tools, such as Freesurfer.

In summary, we developed for the first time an explainable machine learning pipeline, based on various combinations of input features from baseline, to predict expected lateral ventricular enlargement within HD progression and to stratify individuals into groups with more similar prognosis. Our study shows that ventricular volume is an informative biomarker of accelerated brain atrophy in HD progression. We observed that the inclusion of imaging-derived features resulted in significant improvements in predictive accuracy. Our findings suggest that the proposed stratification model could serve as a complementary enrichment tool along with the HD-ISS, after recruited participants are stratified with respect to their PIN scores. This study provides insights into potential surrogate endpoints for experimental therapeutics in HD and could inform future clinical trial design.

## Funding

TRACK-HD and TrackON-HD were supported by the CHDI Foundation, a not-for-profit organisation dedicated to finding treatments for Huntington’s disease. IMAGE-HD was supported by CHDI Foundation research agreement A-3433 and the National Health and Medical Research Council (NHMRC) Australia grant 606,650 (N.G.-K.). The PREDICT-HD study was funded by the NCATS and the NIH (NIH; R01—NS040068, U01—NS105509, U01—NS103475).

Data from the PREDICT-HD, TRACK-HD, and IMAGE-HD studies can be accessed through CHDI foundation upon request. The written scripts used for model development and evaluation in this study are available at: https://github.com/mghofrani/HD_prognostic_model.

## CRediT authorship contribution statement

**Mohsen Ghofrani-Jahromi:** Writing – review & editing, Writing – original draft, Visualization, Validation, Methodology, Conceptualization. **Govinda R. Poudel:** Writing – review & editing, Validation, Supervision, Methodology, Conceptualization. **Adeel Razi:** Writing – review & editing, Supervision, Conceptualization. **Pubu M. Abeyasinghe:** Writing – review & editing, Visualization, Supervision, Project administration, Methodology, Data curation. **Jane S. Paulsen:** Writing – review & editing, Visualization, Validation. **Sarah J. Tabrizi:** Writing – review & editing, Validation, Methodology, Conceptualization. **Susmita Saha:** Writing – review & editing, Validation, Supervision, Methodology. **Nellie Georgiou-Karistianis:** Writing – review & editing, Writing – original draft, Validation, Supervision, Project administration, Methodology, Investigation, Funding acquisition, Data curation, Conceptualization.

## Declaration of competing interest

The authors declare that they have no known competing financial interests or personal relationships that could have appeared to influence the work reported in this paper.

## Data Availability

The authors do not have permission to share data.
