# Supplementary material for: Prognostic enrichment for early-stage Huntington’s disease: An explainable machine learning approach for clinical trial
Source: Neuroimage Clin. 2024 Aug 10;43:103650. doi: 10.1016/j.nicl.2024.103650 (PMC11367643; doi:10.1016/j.nicl.2024.103650)
Supplement: Supplementary Data 1 [file mmc1.docx]

**Supplementary Materials for** “**Prognostic Enrichment for Early-Stage Huntington's Disease: An Explainable Machine Learning Approach for Clinical Trial”**

Mohsen Ghofrani-Jahromi, Govinda R. Poudel, Adeel Razi, Pubu M. Abeyasinghe, Jane S. Paulsen, Sarah J. Tabrizi, Susmita Saha, and Nellie Georgiou-Karistianis

# Feature domains and evaluation metrics

**Table 1.** A comprehensive list of features used in each domain. Referring to Figure 2 in the manuscript, the domains are sequentially added to the input features set.

|  | Feature domain | Description |
| --- | --- | --- |
| - | Confounding variables | Age and sex were included in the feature set for all models.  Intracranial volume (ICV) was used only in models that contained imaging derived features. |
| I | CAP | Age × (CAG – 35) |
| II | Cognitive Scores | Symbol Digit Modalities Test (SDMT)  Stroop word reading test (SWR) |
| III | TMS | Total Motor Score subscale of the Unified Huntington’s Disease Rating Scale (UHDRS®) |
| IV | WMH | The volume of white matter hypo-intensities |
| V | Subcortical Volumes | Volumes of cerebellum white matter, cerebellum cortex, thalamus, caudate, putamen, pallidum, brainstem, hippocampus, amygdala, accumbens area, ventral dc, vessel, optic chiasm, and posterior, mid posterior, central, mid-anterior, anterior subfields of the corpus callosum. |
| VI | Ventricular Volumes | Volumes of lateral ventricles, inferior lateral ventricles, 3^rd^ ventricle, 4^th^ ventricle, CSF, and choroid plexus. |
| VII | Cortical Morphometry | Both volumes and thicknesses of bilateral cortical regions including bankssts, caudal anterior cingulate, caudal middle frontal, cuneus, entorhinal, fusiform, inferior parietal, inferior temporal, isthmus cingulate, lateral occipital, lateral orbitofrontal, lingual, medial orbitofrontal, middle temporal, para hippocampal, paracentral, pars opercularis, pars orbitalis, pars triangularis, pericalcarine, postcentral, posterior cingulate, precentral, precuneus, rostral anterior cingulate, rostral middle frontal, superior frontal, superior parietal, superior temporal, supra marginal, frontal pole, temporal pole, transverse temporal, and insula. |

**Table 2.** Prognostic models training, validation, and test results. Size refers to the number of samples and features in each dataset. R^2^ score is the coefficient of determination which measures the goodness of fit. MAE: Mean Absolute Error.

|  | | I | II | III | IV | V | VI | VII |
| --- | --- | --- | --- | --- | --- | --- | --- | --- |
| **Train** | Size | (537, 4) | (537, 6) | (537,7) | (537, 8) | (537,27) | (537, 33) | (537, 101) |
|  | R^2^ | 0.34 ± 0.01 | 0.45 ± 0.01 | 0.48 ± 0.01 | 0.51 ± 0.01 | 0.63 ± 0.01 | 0.66 ± 0.01 | 0.71 ± 0.01 |
|  | MAE | 647.2 ± 14 | 606.4 ± 14 | 595.2 ± 14 | 573.8 ± 13 | 494.4 ± 9.8 | 464.3 ± 8.2 | 431.3 ± 7.4 |
| **Validation** | R^2^ | 0.19 ± 0.12 | 0.32 ± 0.08 | 0.35 ± 0.10 | 0.38 ± 0.09 | 0.49 ± 0.07 | 0.52 ± 0.09 | 0.52 ± 0.08 |
|  | MAE | 705.5 ± 86 | 658.0 ± 95 | 648.9 ± 93 | 628.2 ± 87 | 564.4 ± 73 | 531.2 ± 52 | 534.6 ± 66 |
| **Test** | Size | (56, 4) | (56, 6) | (56, 7) | (56, 8) | (56,27) | (56,33) | (56,101) |
|  | R^2^ | 0.18 | 0.28 | 0.34 | 0.39 | 0.47 | 0.52 | 0.55 |
|  | MAE | 757.46 | 666.78 | 636.19 | 639.99 | 614.15 | 534.11 | 526.55 |

# Volume of White Matter Hypo-intensities on T1-Weighted images

Previous research has also highlighted a robust correlation between T2-weighted WM hyper-intensities and T1-weighted WM hypo-intensities.^1^ To ensure the independence of our modelling approach from additional imaging modalities, we specifically relied on T1-weighted images. Furthermore, T2-weighted scans were only available for a limited number of individuals in this study. Fig.1 illustrates how the ICV-normalised volumes of WM hypo-intensities on the T1-weighted images were distributed across the groups over datasets utilized in this study.

**
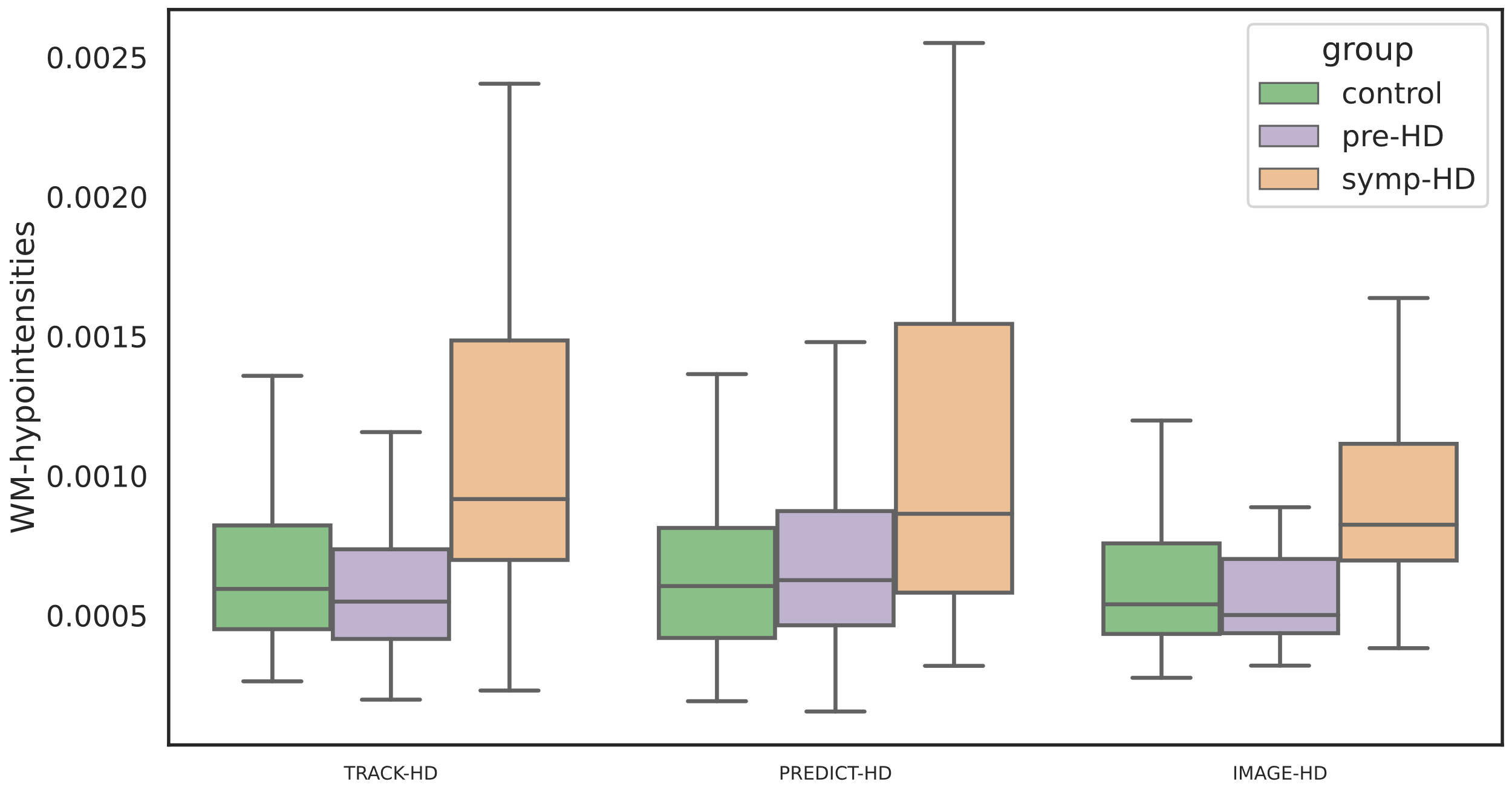
**

**Fig. 1 ICV-normalized volumes of white matter hypo-intensities on T1-weighted images spread across groups and cohorts.**

# Stage-wise comparison of choroid plexus volume

The trend of ICV-normalized enlargement of the choroid plexus is shown below both in the HD individuals (the red line) and healthy controls (the green line).
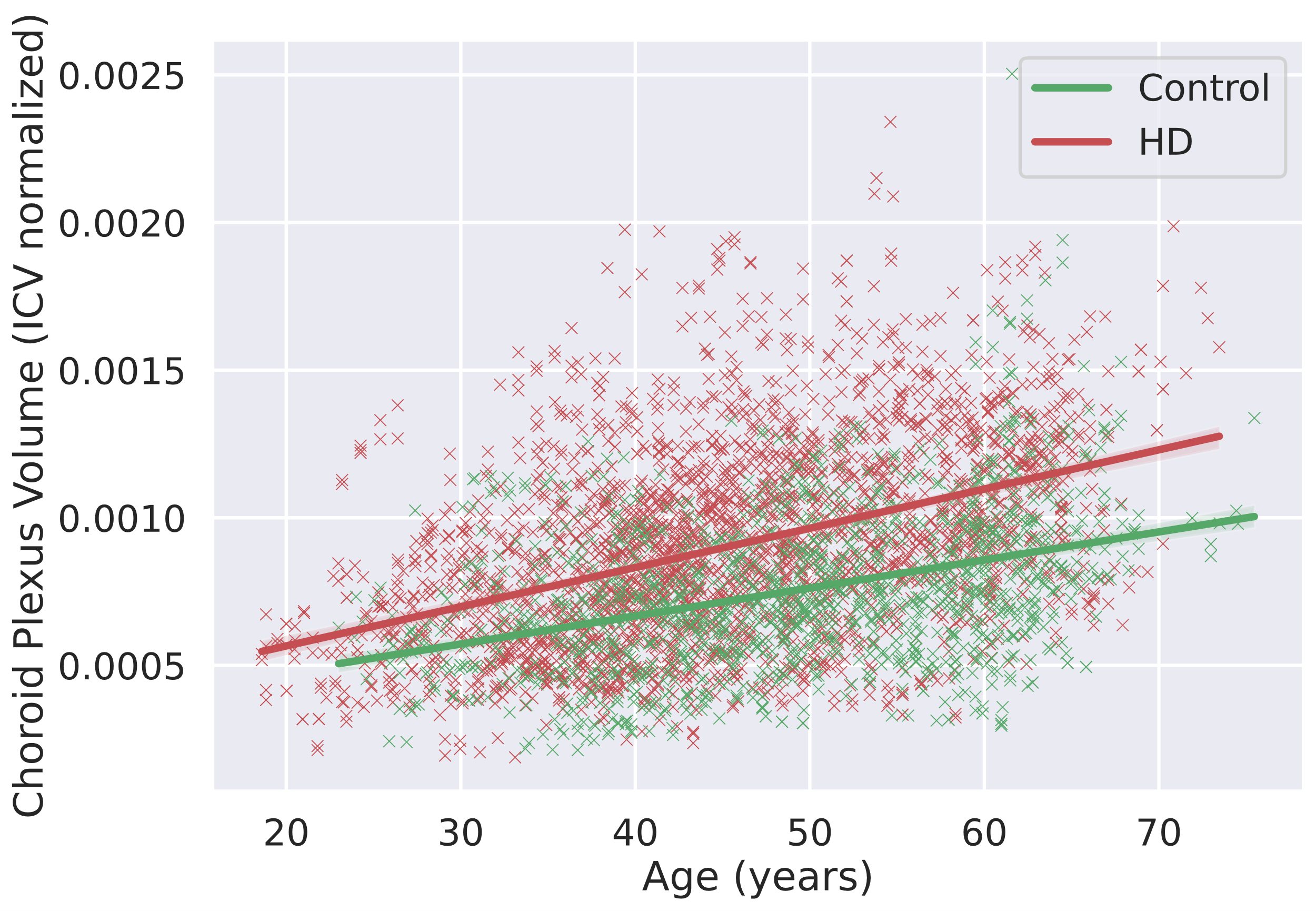


The trend of volumetric enlargement for choroid plexus in HD versus control individuals

We first regressed out the enlargement of the choroid plexus due to normal aging. Then, we used an independent t-test to compare the volume of the choroid plexus at different stages. We observed that the volume at stage 2 is significantly higher than stage 1, and the volume at stage 3 is higher than stage 2. Enlargement of the choroid plexus has been reported in other studies including depression,^2^ complex regional pain syndrome (CRPS),^3^ schizophrenia,^4^ ALS^5^, multiple sclerosis,^6^ and MCI.^7^ While the enlargement in choroid plexus is not specific to HD, its inclusion as one of the inputs to our random forest models increases the accuracy.


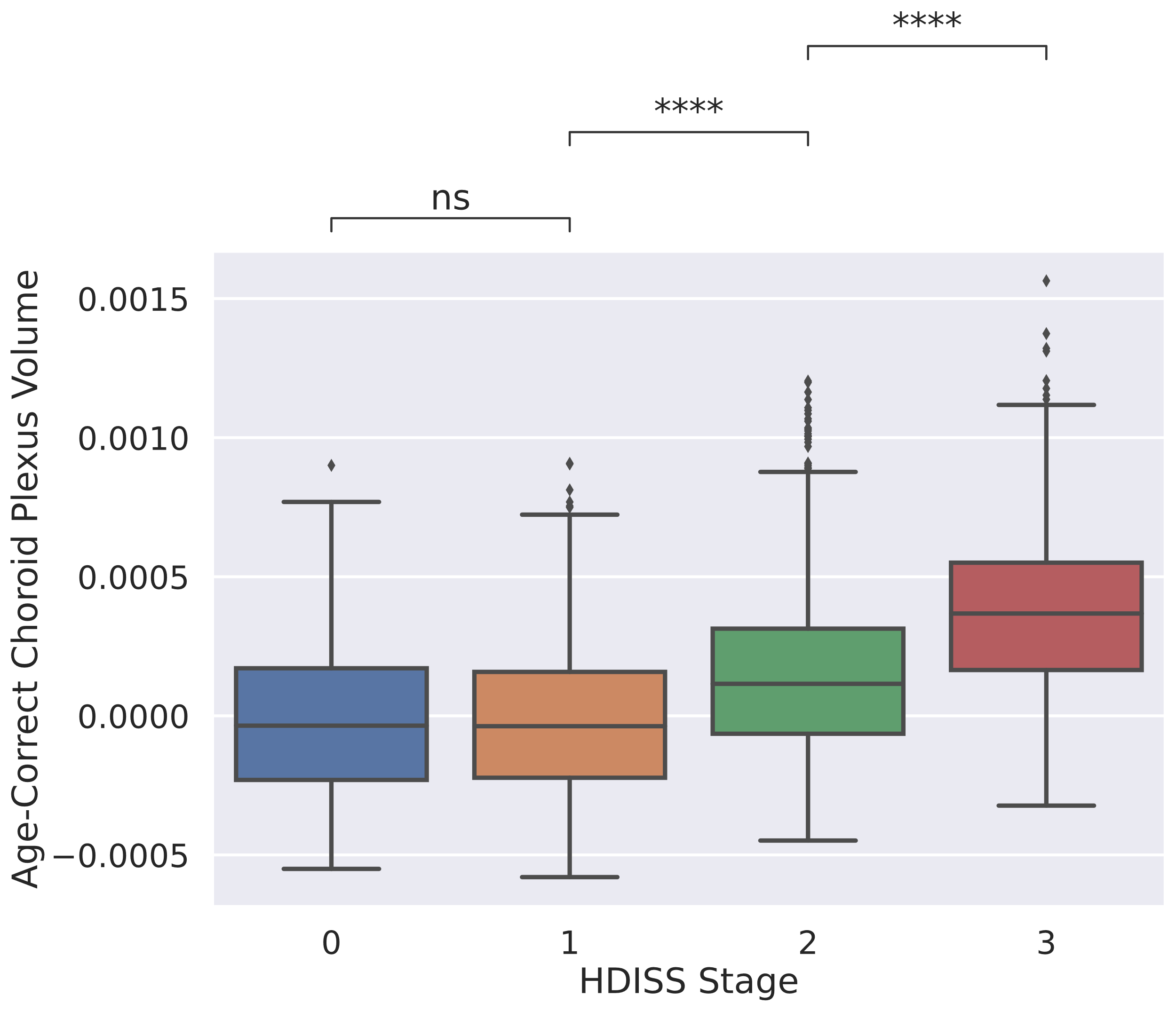


Stage-wise comparison of choroid plexus volume.

# Distribution of lateral ventricular enlargement rate values

A detailed depiction of ground truth LVER values across HD-ISS stages is given in Fig. 2. The red line represents the stratification threshold of 750 mm^3^/year.

The stratification model aims to identify individuals situated to the right of the red line, i.e., those who are the fast progressors and are prioritized for inclusion in clinical trials for screening in. Similarly, prognostic enrichment in a clinical trial involves excluding individuals who have moderate LVERs, because they contribute less to sensitivity to treatment effect.

Inevitably, the stratification model cannot very well discriminate between those samples that are very close to the red line and to its left and those that are very close to the red line and to its right side. Additional details are available in the subsequent section related to optimal selection of stratification threshold.

With outliers disregarded, the range of LVER values extends over two orders of magnitude, from 100 to 10,000, due to the quadratic-like pattern of ventricular enlargement across the lifespan.


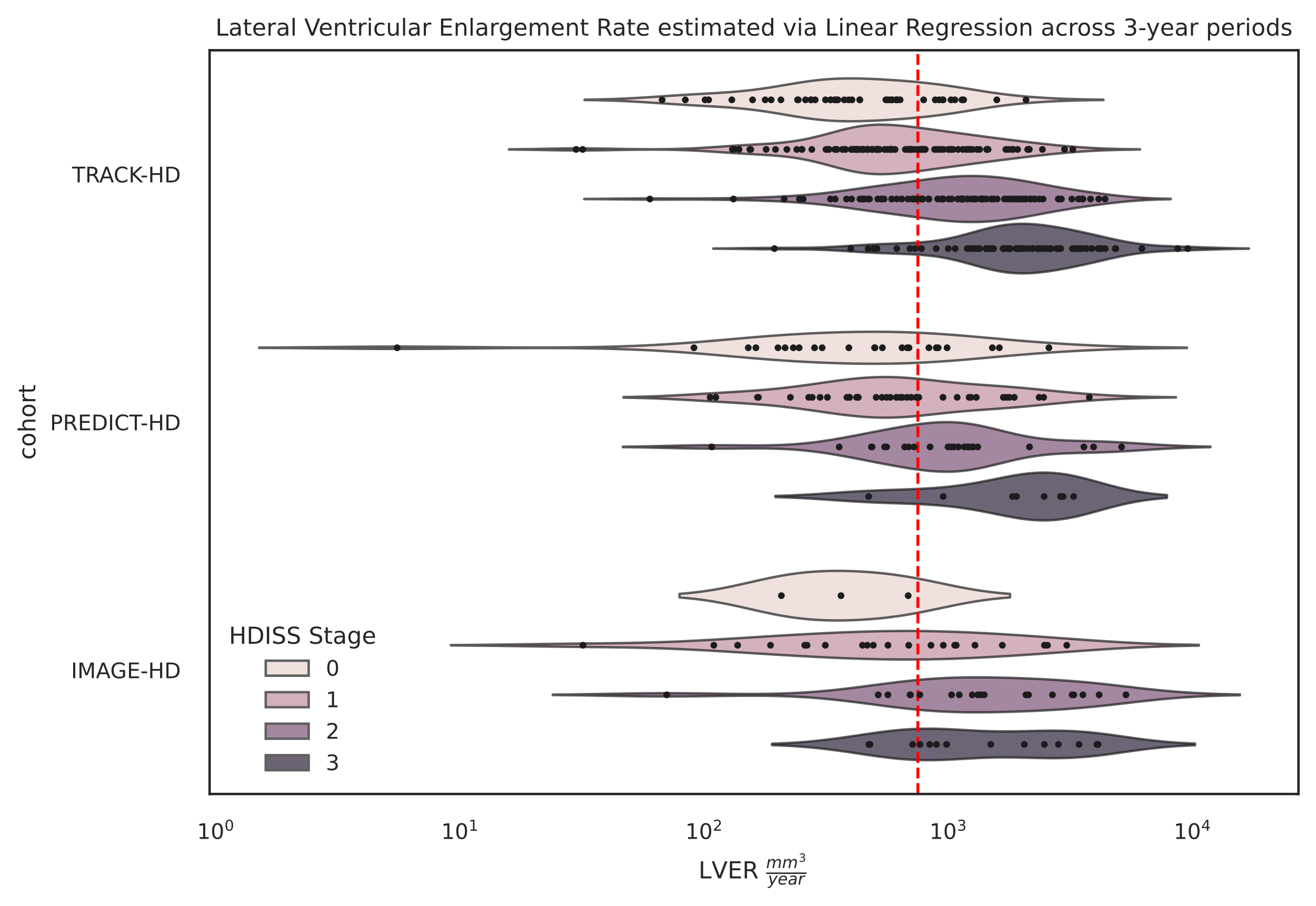


**Fig. 2 Values of ground truth lateral ventricular enlargement rate (LVER) spread over HD-ISS stages.**

# Alternative prognostic model based on regularised regression

Both feature selection and hyperparameter tuning processes were carried out using only the training dataset, with the test set remaining untouched. The hyperparameters of the random forests included the maximum number of leaf nodes, minimum number of samples in each individual decision tree, and the maximum number of samples and features to be used for training. Prognostic models based on regularised regression using least angle regression (LARS), shown in Fig. 3, exhibited a similar pattern of mean absolute error reductions to that observed in random forests. This replication of the MAE reduction trend serves to validate the feature rankings obtained through the implementation of random forests.

Nevertheless, in our pipeline development, random forests were preferred to regularized linear regression since their Shapley explanations were highly aligned with findings in Huntington’s disease clinical research literature. This could be described by the fact that LARS, which aims to find a sparse solution by adding an L1 penalty to the loss function, encourages some of the coefficients to be exactly zero.^8^ As a result, during the optimization process, certain features might receive higher coefficients and contribute more to the model’s output, not because they have a strong physical meaning or direct relationship with the target variable, but rather due to their ability to reduce the overall loss and meet the regularization constraint.

Shapley explanations of the prognostic model based on LARS is shown in Fig. 4. The diagrams indicate that in certain regions, such as the left hemisphere middle temporal lobe, a thicker cortex corresponds to more severe disease progression, which contradicts our existing understanding of cortical thinning associated with neurodegeneration.


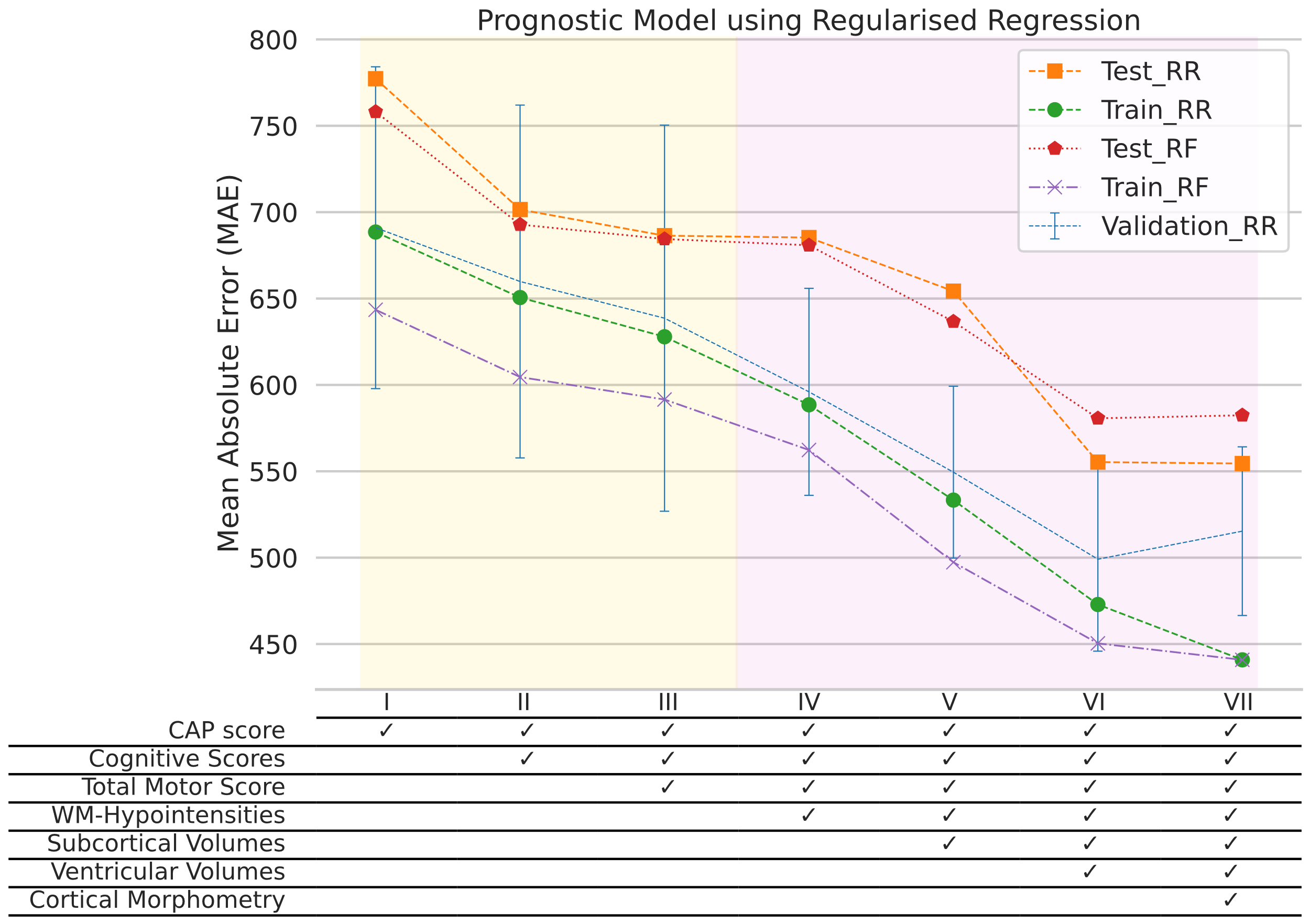


**Fig. 3 Prognostic models based on regularized regression (RR) and random forests (RF).** Similar trends of error reduction seen for random forests were repeated as features were sequentially integrated to the input features set.


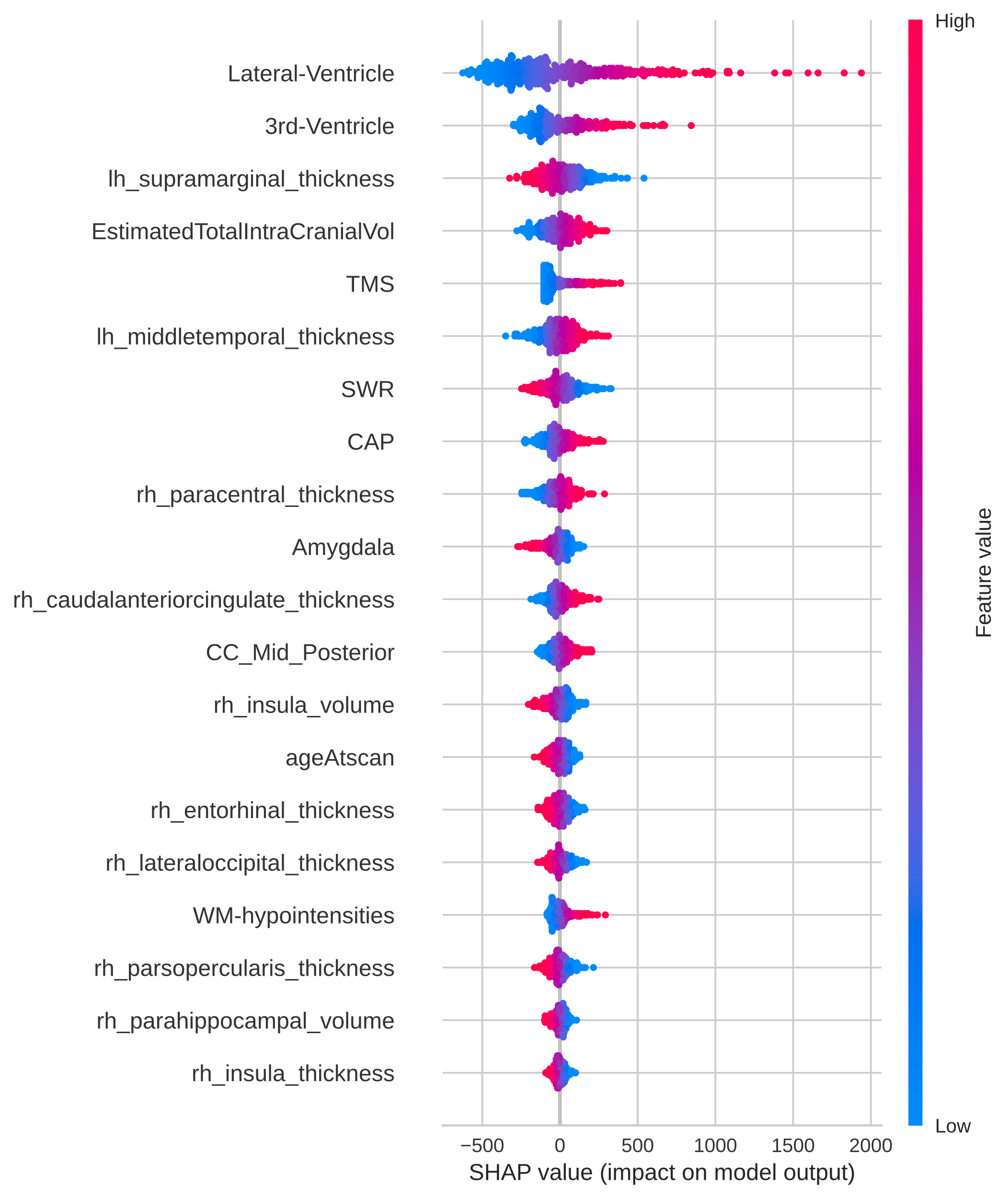


**Fig. 4 Shapley explanation of the prognostic model based on regularized regression.**

In contrast, random forests impose no prior assumptions on the input data. Shapley explanations derived from the proposed random forest model regarding cortical thinning in the lateral occipital lobes align with findings from clinical studies. Specifically, the presence of blue sections on the right side of the diagrams supports the understanding that thinner cortical regions are associated with advanced progression of Huntington’s disease.^9,10^


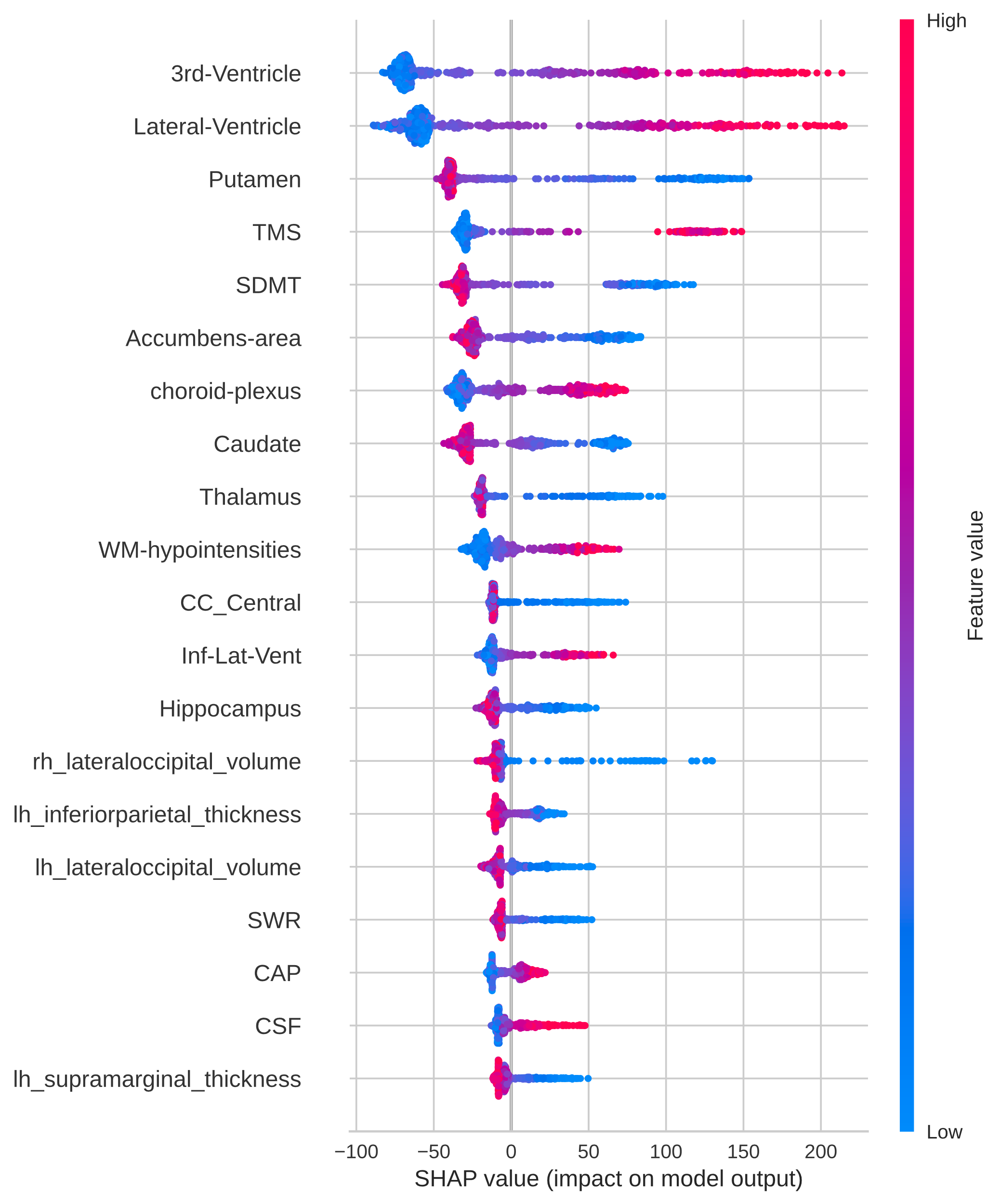


**Fig. 5 Shapley explanation of the proposed prognostic model based on random forests**

# Optimal selection of stratification threshold

The suggested threshold of 750 mm³/year strikes a balance between specificity and sensitivity, potentially serving as an optimal choice for achieving the desired specificity-sensitivity trade-off. However, in a clinical trial the threshold can be tailored to prioritize certain aspects of participant recruitment. If the primary objective is to identify and include individuals with fast disease progression, a lower threshold might be preferred, as it would capture a greater number of individuals exhibiting rapid deterioration. On the other hand, if the focus is on excluding individuals with moderate progression, a higher threshold would be more appropriate, as it would filter out those who are not experiencing significant deterioration.


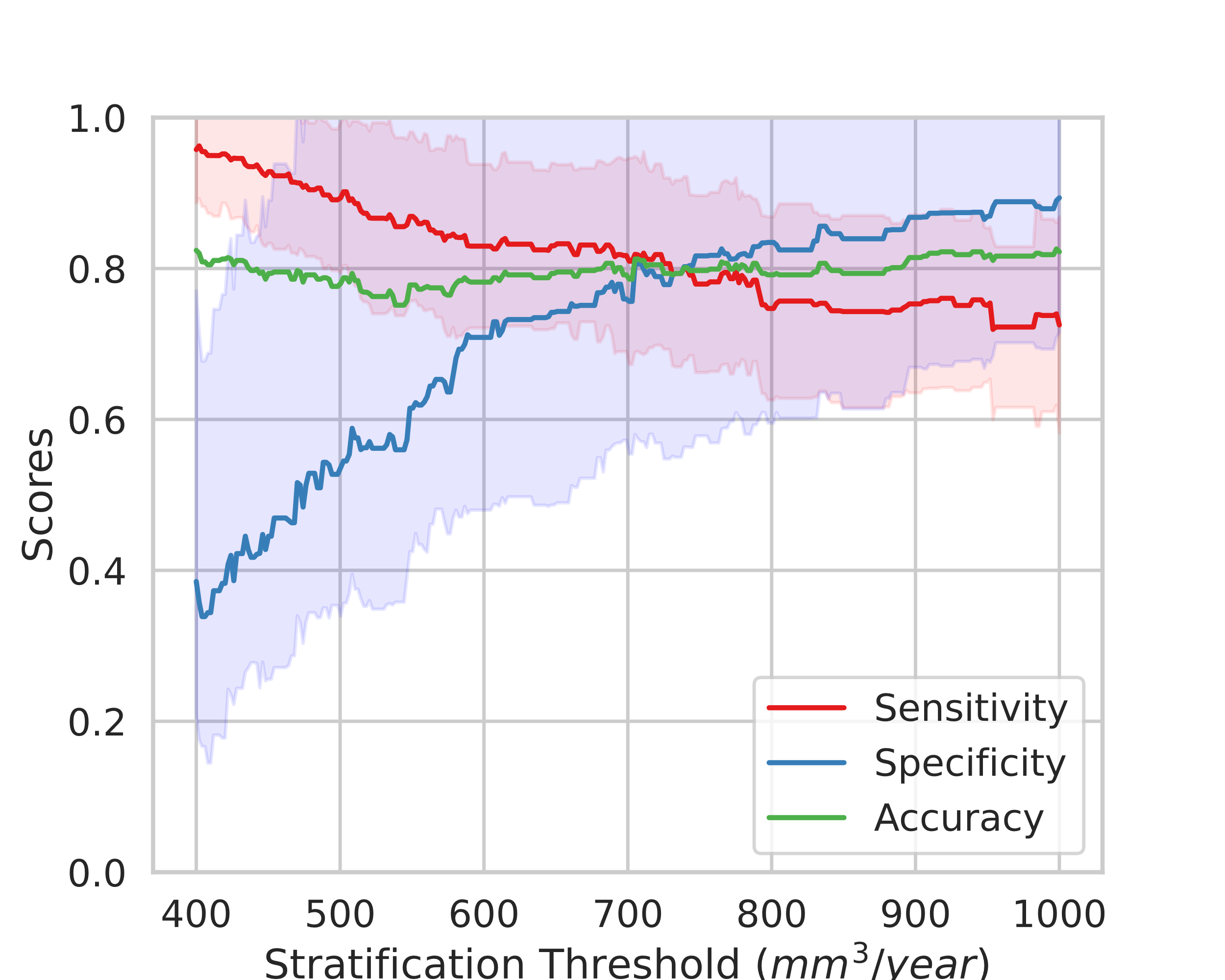


**Fig. 6** **The effect of threshold variation on evaluation metrics for stratification model.** The stratification model is essentially a classifier that predicts the likelihood of moderate or fast ventricular enlargement based on a specified threshold. The choice of threshold can be adjusted to meet clinical requirements, but it should be noted that threshold values that are far from the median can result in imbalanced classes and low specificity or sensitivity.

# Confusion matrices for subgroups

We conducted training and testing using leave-one-out cross-validation (LOOCV), exclusively based on imaging features.

**Input feature set = {Sex, Age, Ventricular Volumes, White Matter Hypo-intensities volume, Subcortical Volumes, ICV}**

This section gives a description of how modifying the stratification threshold affects the correct identification of:

- pre-HD moderate progressors,
- pre-HD fast progressors,
- symp-HD moderate progressors,
- and symp-HD fast progressors.

Our dataset consisted of 153 symp-HD and 370 pre-HD baseline samples. Among the 370 pre-HD samples, 139 had actual values of LVER higher than the threshold of 750mm^3^/year. We observed that among the 139 pre-HD fast progressors, 48 individuals were incorrectly classified as moderate progressors.

We noticed that while the model successfully identified 91 of pre-HD fast progressors, it also exhibited limitations in accurately distinguishing them from moderate progressors. Identifying symp-HD fast progressors is a straightforward task. The model had a sensitivity of 92% in identifying them. As seen in the table below, the specificity of the model for the purpose of excluding pre-HD moderate progressors is 86%, however its sensitivity to identify pre-HD fast progressors is 65%. Results are summarised in Table 2.

By reducing the stratification threshold from 750mm^3^/year to 600mm^3^/year, the model became more sensitive at the cost of losing some specificity. Table 3 illustrates confusion matrices.

On the other hand, increasing the stratification threshold from 750mm^3^/year to 900mm^3^/year led to a more specific stratification model at the cost of sensitivity, shown in Table 4.

**Table 2. Confusion matrix for the stratification model with a threshold of 750 mm^3^/year**

| **pre-HD** (N = 370) | **symp-HD** (N = 153) |
| --- | --- |
| \|  \| \| Actual \| \| \| --- \| --- \| --- \| --- \| \| Fast \| Moderate \| \| Predicted \| Fast \| 91 \| 32 \| \| Moderate \| 48 \| 199 \|   Sensitivity = tp/(tp+fn) = 91/139 = 65%  Specificity = tn/(tn+fp) = 199/231 = 86% | \|  \| \| Actual \| \| \| --- \| --- \| --- \| --- \| \| Fast \| Moderate \| \| Predicted \| Fast \| 123 \| 12 \| \| Moderate \| 10 \| 8 \|   Sensitivity = tp/(tp+fn) = 123/133 = 92%  Specificity = tn/(tn+fp) = 8/20 = 40% |

**Table 3. Confusion matrix for the stratification model with a threshold of 600 mm^3^/year**

| **pre-HD** (N = 370) | **symp-HD** (N = 153) |
| --- | --- |
| \|  \| \| **Actual** \| \| \| --- \| --- \| --- \| --- \| \| Fast \| Moderate \| \| **Predicted** \| Fast \| 125 \| 44 \| \| Moderate \| 54 \| 147 \|     Sensitivity = tp/(tp+fn) = 125/179 = 70%  Specificity = tn/(tn+fp) = 147/191 = 76% | \|  \| \| **Actual** \| \| \| --- \| --- \| --- \| --- \| \| Fast \| Moderate \| \| **Predicted** \| Fast \| 133 \| 11 \| \| Moderate \| 5 \| 4 \|   Sensitivity = tp/(tp+fn) = 133/138 = 96%  Specificity = tn/(tn+fp) = 4/15 = 27% |

**Table 4. Confusion matrix for the stratification model with a threshold of 900 mm^3^/year**

| **pre-HD** (N = 370) | **symp-HD** (N = 153) |
| --- | --- |
| \|  \| \| **Actual** \| \| \| --- \| --- \| --- \| --- \| \| Fast \| Moderate \| \| **Predicted** \| Fast \| 70 \| 18 \| \| Moderate \| 49 \| 233 \|   Sensitivity = tp/(tp+fn) = 70/119 = 59%  Specificity = tn/(tn+fp) = 233/ 251 = 93% | \|  \| \| **Actual** \| \| \| --- \| --- \| --- \| --- \| \| Fast \| Moderate \| \| **Predicted** \| Fast \| 114 \| 12 \| \| Moderate \| 10 \| 17 \|   Sensitivity = tp/(tp+fn) = 114/124 = 92%  Specificity = tn/(tn+fp) = 17/29 = 58% |

We repeated our model training with a threshold of 750 mm^3^/year while also including additional features such as genetic, cognitive, and motor assessments as well as composite scores in the input feature set.

**Input feature set = {Sex, Age, CAP, TMS, SDMT, SWR, cUHDRS, PIN, Ventricular Volumes, White Matter Hypo-intensities volume, Subcortical Volumes, ICV}**

We hypothesized that by incorporating these additional biomarkers, the model would have a better understanding of the disease stages, ultimately leading to a reduction in incorrectly classified pre-HD fast progressors. However, the number of incorrectly classified pre-HD fast progressors actually increased when compared to our previous model that solely utilized imaging features. In other words, in order to identify pre-HD fast progressors, the stratification model finds the imaging-derived features more effective than conventional biomarkers of disease stage. This is in line with the notion that imaging features carry more relevant and predictive information regarding disease progression in the pre-HD stage. For reference, compare incorrectly classified pre-HD fast progressors in Table 2 and Table 5.

**Table 5. Confusion matrix for the stratification model with a threshold of 750 mm^3^/year while conventional biomarkers of disease progression were added to the input feature set**

| **pre-HD** (N = 370) | **symp-HD** (N = 153) |
| --- | --- |
| \|  \| \| **Actual** \| \| \| --- \| --- \| --- \| --- \| \| Fast \| Moderate \| \| **Predicted** \| Fast \| 84 \| 28 \| \| Moderate \| **55** \| 203 \|     Sensitivity = tp/(tp+fn) = 84/139 = 60%  Specificity = tn/(tn+fp) = 203/231 = 88% | \|  \| \| **Actual** \| \| \| --- \| --- \| --- \| --- \| \| Fast \| Moderate \| \| **Predicted** \| Fast \| 127 \| 15 \| \| Moderate \| 6 \| 5 \|   Sensitivity = tp/(tp+fn) = 127/133 = 95%  Specificity = tn/(tn+fp) = 5/20 = 25% |

# Modelling Huntington’s disease continuum

A frequently employed method for computational modelling of disease progression in neurodegenerative conditions involves conceptualizing the disease trajectory as comprising two separate and discernible stages. During the early phase, it is presumed that patients will have biomarkers that are comparable to those of healthy individuals. In the second state of the disease course, patients' biomarker trajectories are expected to deviate from those of healthy individuals, and cut-off values are defined for the hallmark biomarkers of the disease under study.

In this context, an example is given in Fig. 7A where the reduction in the putamen volume within Huntington’s disease continuum is significant when compared with healthy controls. However, this may not be the case for cortical thickness measurements. In Fig. 7B, a comparison of insular thickness among the three groups is depicted. The measured insular thickness of pre-HD individuals is significantly higher than that of the symp-HD group. However, in comparison to healthy controls, the pre-HD group does not exhibit a thinner insular cortex, but rather the opposite - a significantly thicker insular cortex. Table 6 presents the baseline demographic characteristics of healthy control individuals.

**Table 6. Demographic characteristics of healthy control individuals at baseline used also for training normal aging digital twin**.

| Cohort | N | % Female | Age | LVER |
| --- | --- | --- | --- | --- |
| TRACK | 145 | 56 | 45.9 ± 10.1 | 458 (735) |
| PREDICT | 54 | 61 | 48.8 ± 10.1 | 338 (520) |
| IMAGE | 29 | 69 | 43.7 ± 13.4 | 404 (605) |
| Pooled Data | 228 | 59 | 46.3 ± 10.6 | 423 (673) |

| **A** | 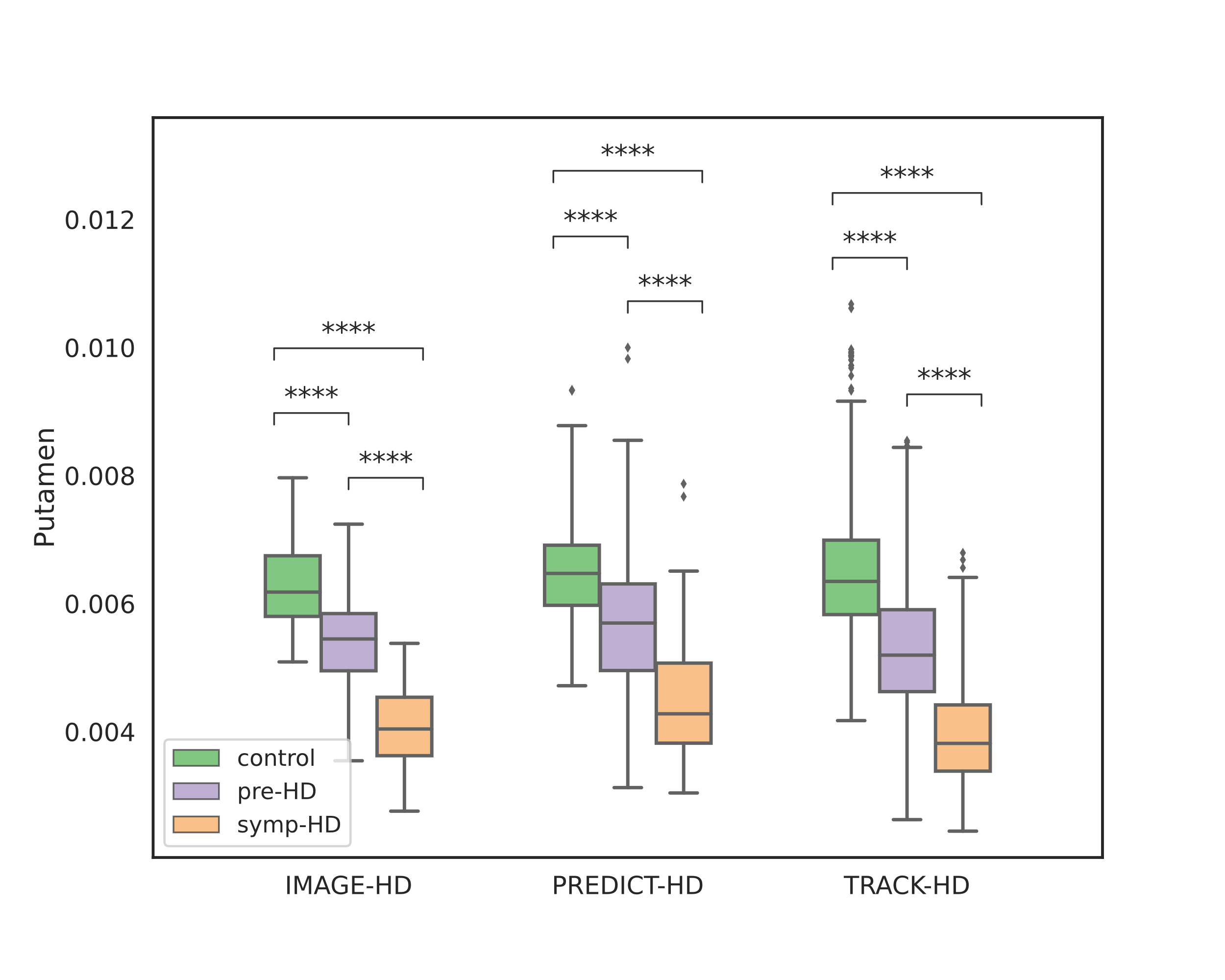 |
| --- | --- |
| **B** | 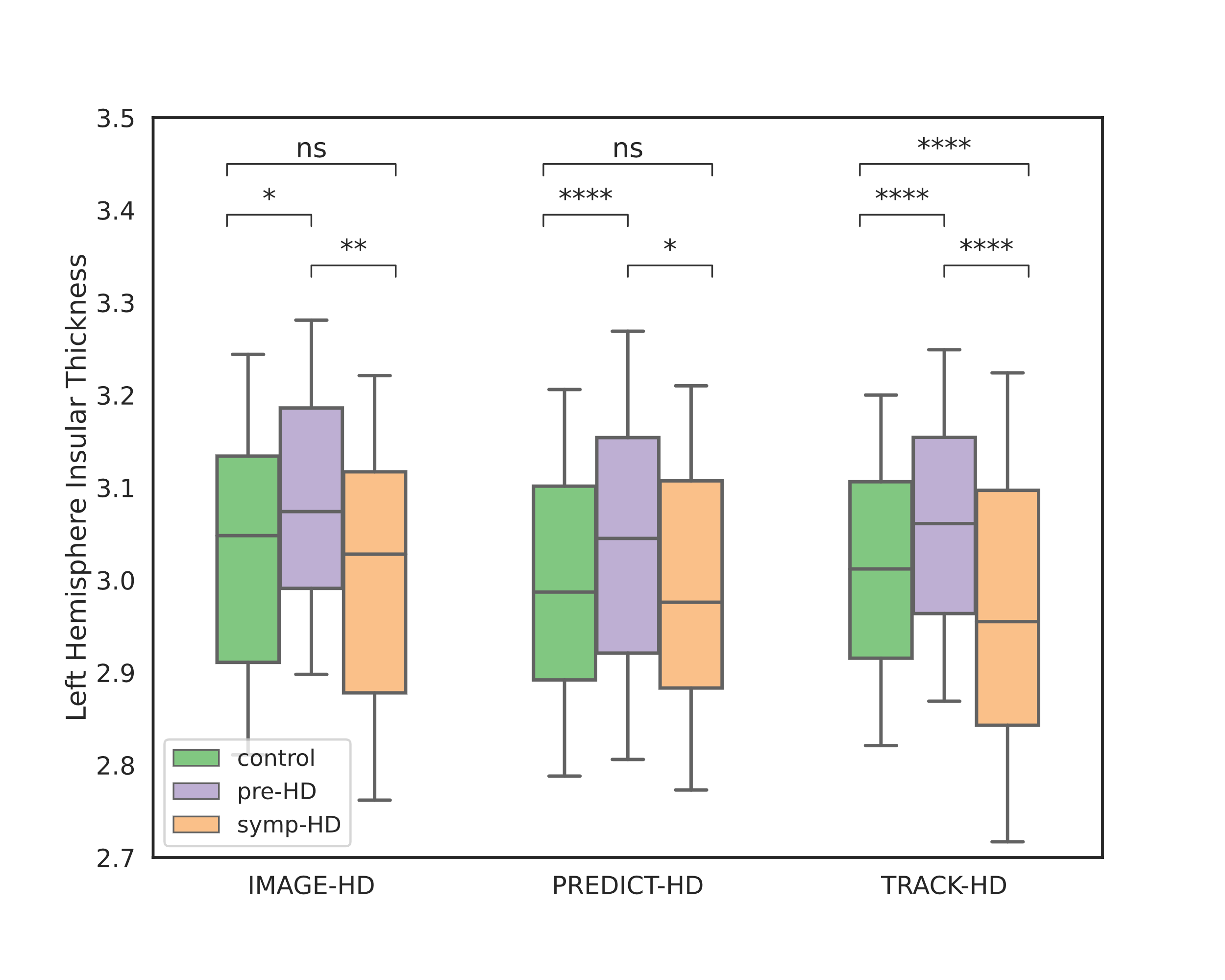 |

**Fig. 7** **(A)** The reduction in putamen volume during the course of Huntington's disease is apparent in all three pairwise group comparisons across all cohorts. **(B)** The higher insular cortical thickness observed in pre-HD groups compared to healthy controls is thought to be due to neurodevelopmental factors associated with Huntington’s disease.

# Decomposing age-related and pathogenic ventricular enlargement

Ventricular enlargement is not only non-specific to HD but also common in normal aging. The use of SHAP paves the way for confirming that the models’ “understanding” of the underlying pathogenic process aligns with previous evidence from brain imaging studies of HD. This provides greater confidence in the accuracy of the models' predictions. Nonetheless, it is important to also account for the impact of normal aging on the predicted values of ventricular enlargement. Therefore, we trained two prognostic models of lateral ventricular enlargement for each individual: one model trained with imaging-derived features from other HDGECs and another using the same features but derived from healthy controls.

Illustrated in Fig. 8, each dark-colored arrow shows the projected value of lateral ventricles after one year, whereas light-colored arrows indicate the contribution of normal aging to the projected values. In other words, this technique involves constructing two distinct digital twins for each individual in order to distinguish the component of ventricular enlargement which is attributable to normal aging from the pathogenic component. The characteristics of healthy controls are summarized in Table 6.


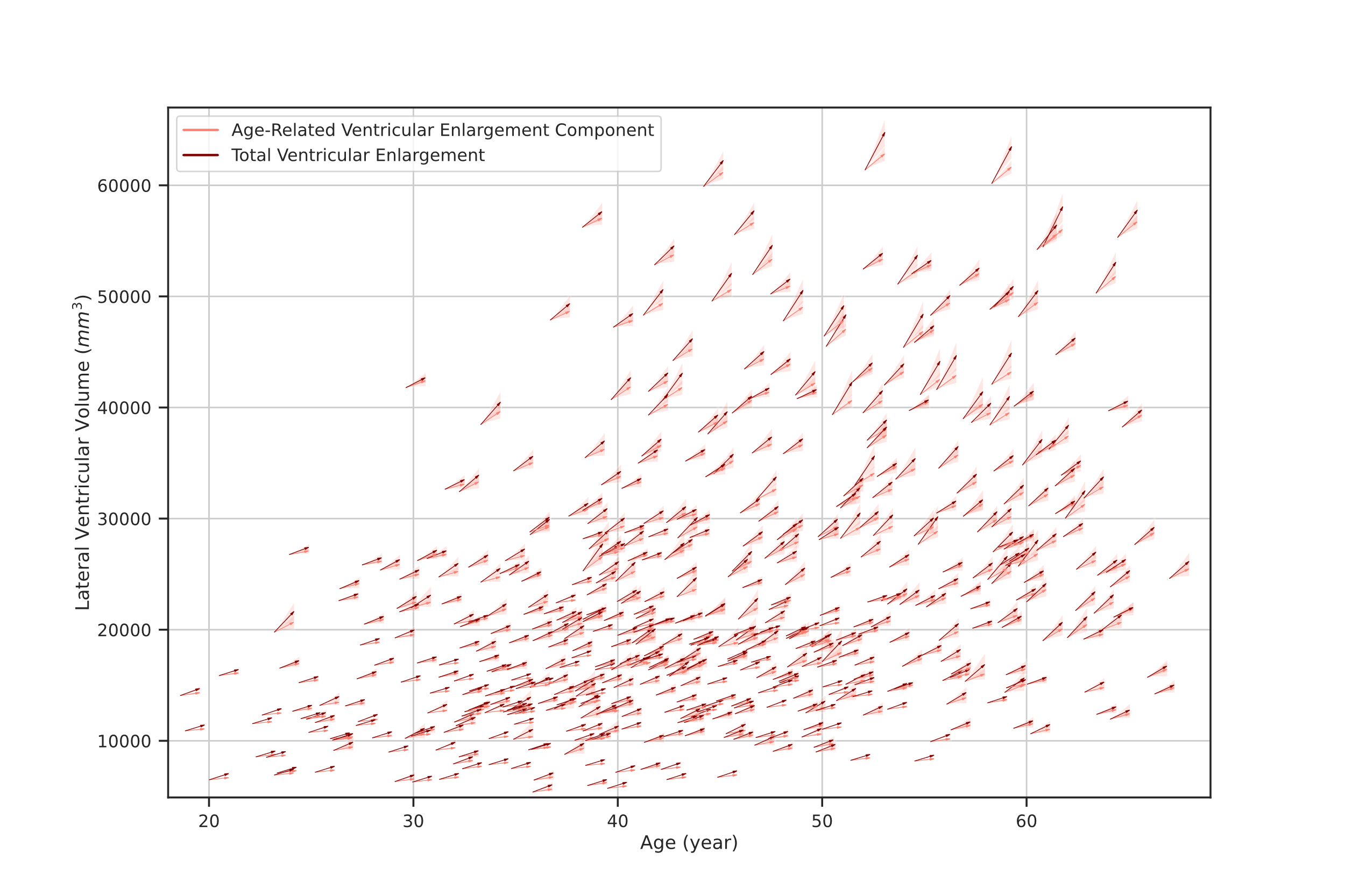


**Fig. 8 Decomposition of lateral ventricular enlargement into pathogenic and normal aging components.** The predicted ventricular expansion after one year is represented by the dark arrows, while the pale-coloured arrows indicate the portion of enlargement attributed to normal aging. The shades of colours highlight the prediction intervals obtained from individual estimators in the random forests.

# Feature rankings of the stratification model trained with non-imaging features

Fig. 9 illustrates the Shapley explanations for a stratification model that has been trained on non-imaging features. The results indicate that PIN and CAP were the most decisive features, while total functional capacity (TFC) had the lowest importance ranking.

Normalized Prognostic Index: $PIN_{HD} =\frac{\left( 51 \times TMS + (-34) \times SDMT + 7 \times Age \times(CAG - 34) \right)-883}{1044}$

Genetic load: CAP = Age × (CAG – 35)

cUHDRS = $10 +\frac{TFC - 10.4}{1.9} -\frac{TMS-29.7}{14.9} +\frac{SDMT-28.4}{11.3} +\frac{SWR - 66.1}{20.1}$


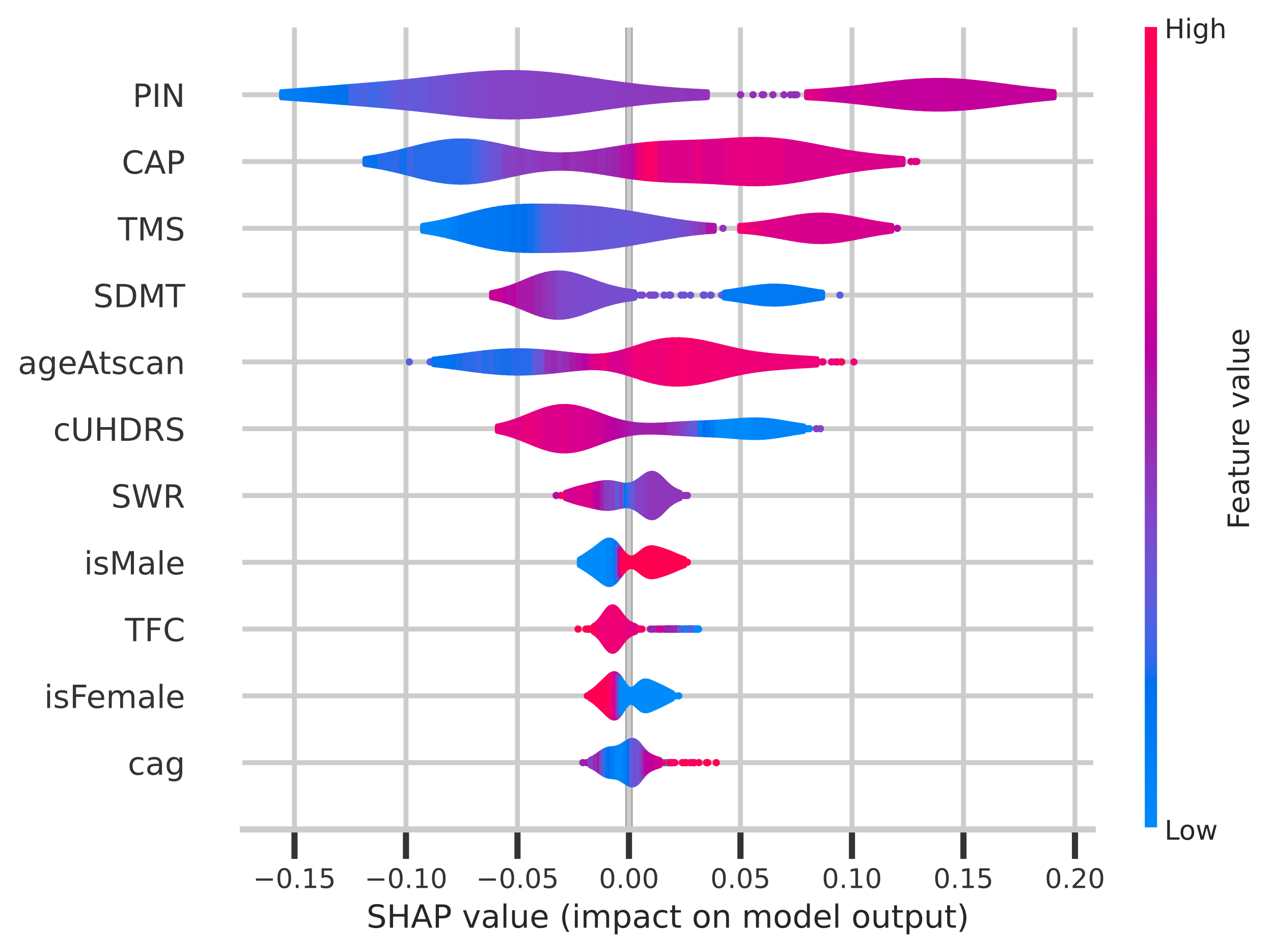


**Fig. 9 Feature importance of genetic, cognitive, motor, and composite scores ranked in a trained stratification model.**

# Model Validation Experiments

In order to ensure that the proposed models are not biased towards a specific cohort, we conducted several experiments, listed in Table 7, using LOOVC. Stratification models were trained using features similar to prognostic model VII.

**Input feature set = {Sex, Age, CAP, TMS, SDMT, SWR, Ventricular Volumes, White Matter Hypo-intensities volume, Subcortical Volumes, ICV, Cortical Thickness, Cortical Volumes}**

IMAGE had the best accuracy among the cohorts, which could be due to the fact that, compared to the other cohorts, IMAGE includes pre-HD individuals with lower TMS, making it easier for the stratification model to distinguish between fast and moderate progressors.

By combining data from multiple cohorts (Exp #5 to Exp #8), the model was able to compensate for discrepancies between the cohorts. For instance, combining PREDICT with IMAGE or TRACK leads to improved predictive performance.

Fig. 10 shows how misclassified samples are distributed across the stratification threshold.

**Table 7. The performance of the stratification model evaluated using different cohorts:** Each experiment involves leave-one-out cross-validation using the specified dataset. The term "noisy" refers to a dataset where 1.5T MRI scans were not excluded.

| Exp # | Data (Train & Validation) | N | LOOCV Accuracy |
| --- | --- | --- | --- |
| 1 | IMAGE | 64 | 0.84 |
| 2 | PREDICT | 112 | 0.77 |
| 3 | PREDICT (Noisy) | 367 | 0.76 |
| 4 | TRACK | 347 | 0.79 |
| 5 | IMAGE + PREDICT | 176 | 0.82 |
| 6 | IMAGE + TRACK | 411 | 0.80 |
| 7 | TRACK + PREDICT | 459 | 0.79 |
| 8 | TRACK + IMAGE + PREDICT | 537 | 0.80 |
| 9 | TRACK + IMAGE + PREDICT (Noisy) | 783 | 0.80 |


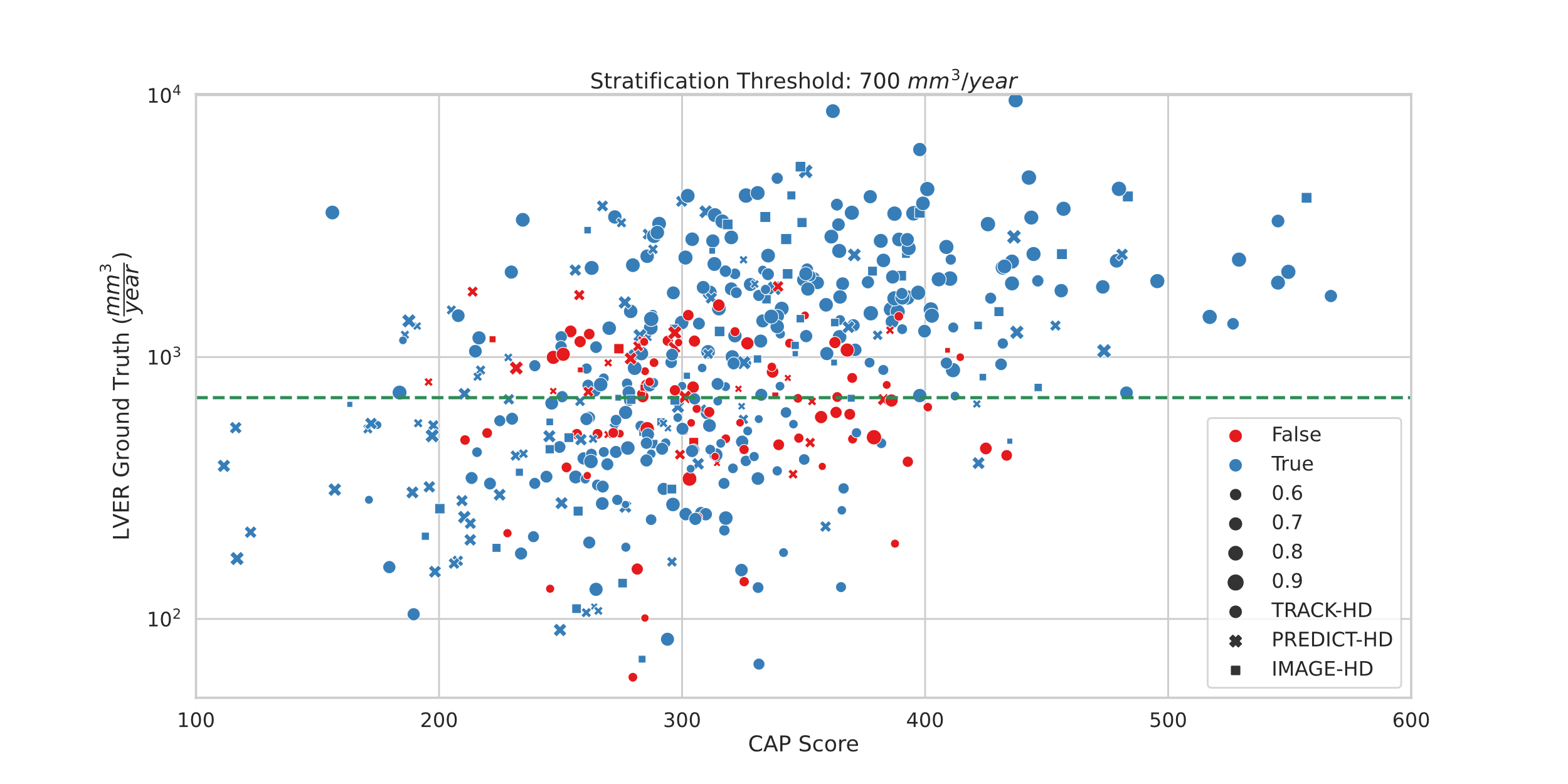


**Fig. 10 Leave-one-out cross validation for the combined dataset.** The vertical axis, in logarithmic scale, represents the ground truth, i.e., lateral ventricular enlargement rate (LVER) calculated via linear regression analysis using longitudinal dataset. Within each iteration in a leave-one-out cross validation, each individual’s data at baseline is tested with a model trained using all other samples. The random forest classifier had to determine whether the anticipated LVER for an individual is below or over the specified threshold of 700 mm^3^/year. 20% of the individuals, shown in red, are misclassified by the stratification model. The size of markers represents the certainty of the model in its predictions. Points which are close to the threshold are more difficult to classify.

We performed cross-study comparisons for the volume of the lateral-ventricle both before and after intracranial volume normalisation. We did not find any significant differences.

| 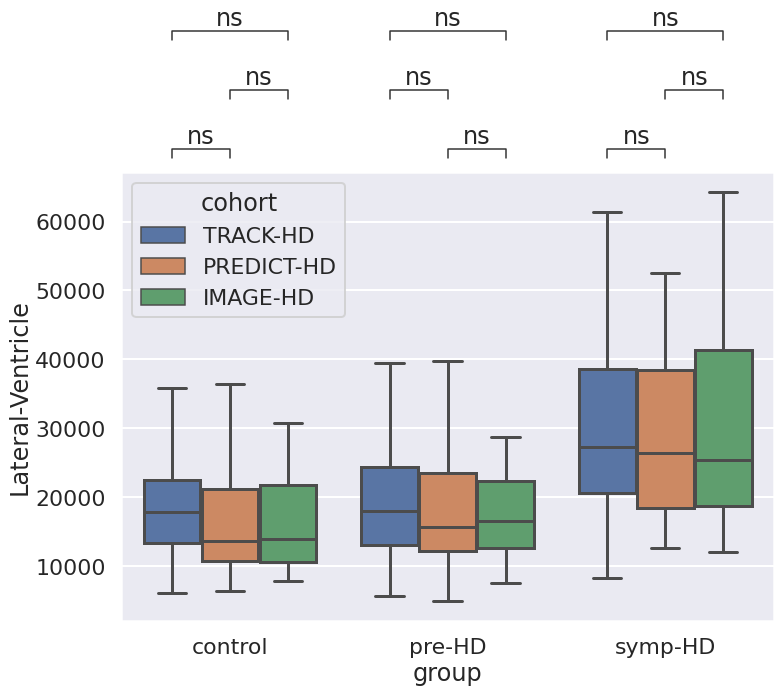 | 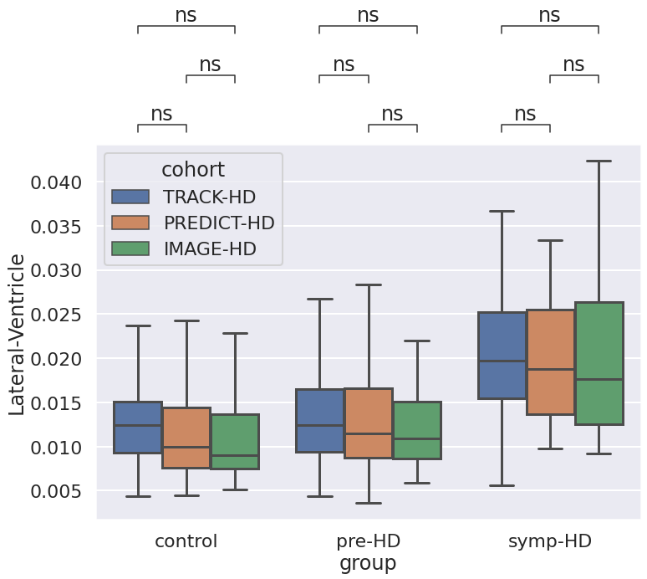 |
| --- | --- |
| **Before ICV-normalistion** | **After ICV-normalisation** |

p-value annotation for ns: 5.00e-02 < p <= 1.00e+00

# Out-of-Sample Testing

We trained and tested the stratification model using dissimilar cohorts to ensure the out-of-sample generalization capability of the proposed stratification model.

Shown in Table 8, in each experiment, we tested the model using a cohort unseen by the model during its training. By not excluding 1.5 Tesla MRI scans from the PREDICT, the accuracy of the model was not severely impacted, which highlights the robustness of random forests to noisy measurements.

**Table 8. Out-of-sample testing for the stratification model.**

| Exp# | Training Set | N | Test Set | Accuracy | Average |
| --- | --- | --- | --- | --- | --- |
| 10 | IMAGE | 64 | PREDICT | 0.73 | 0.74 |
| 11 |  |  | PREDICT (Noisy) | 0.72 |  |
| 12 |  |  | TRACK | 0.76 |  |
| 13 |  |  | TRACK + PREDICT | 0.76 |  |
| 14 | PREDICT | 112 | IMAGE | 0.88 | 0.82 |
| 15 |  |  | TRACK | 0.79 |  |
| 16 |  |  | TRACK + IMAGE | 0.81 |  |
| 17 | PREDICT (Noisy) | 367 | IMAGE | 0.89 | 0.82 |
| 18 |  |  | TRACK | 0.77 |  |
| 19 |  |  | TRACK + IMAGE | 0.79 |  |
| 20 | TRACK | 347 | IMAGE | 0.91 | 0.82 |
| 21 |  |  | PREDICT | 0.79 |  |
| 22 |  |  | PREDICT (Noisy) | 0.75 |  |
| 23 |  |  | PREDICT + IMAGE | 0.84 |  |
| 24 | IMAGE + PREDICT | 176 | TRACK | 0.79 |  |
| 25 | IMAGE + PREDICT (Noisy) | 431 |  | 0.77 |  |
| 26 | IMAGE + TRACK | 411 | PREDICT | 0.78 |  |
| 27 |  |  | PREDICT (Noisy) | 0.75 |  |
| 28 | TRACK + PREDICT | 459 | IMAGE | 0.88 |  |
| 29 | TRACK + PREDICT (Noisy) | 719 |  | 0.88 |  |

Moreover, we included site information as input covariate in our random forest model to investigate its impact on the model's output. We did not observe any significant changes. In fact, in our SHAP analysis, 'site' was among the 10 least important features (out of ~170) that affected the output.

# Case study

The biomarkers of disease progression over a 7-year period for a female pre-HD individual in the unseen test set are shown in Fig. 11. The data involves time points at ages of: t_1_=39.4, t_2_=40.4, t_3_=41.4, t_4_=42.4, t_5_=44, t_6_=44.8, and t_7_=45.8 years. The issue of ceiling and floor effects in TFC and TMS, as well as inconsistent fluctuations in SDMT and SWR are noticeable, whereas the composite scores are less affected by these problems.

The last two panels present the output of the proposed prognostic and stratification models, respectively. At most of the time points, the actual lateral ventricular volume in the subsequent brain scan falls within the range of values predicted by the prognostic model. Here, the participant is consistently classified as a fast progressor at all time points because the certainty of the model about LVER exceeding the threshold of 750 mm^3^/year is consistently above 0.5 and shows continuous growth longitudinally. Subject-specific SHAP values of the model at t_1_ and t_4_ are provided in the Figures 12 and 13.


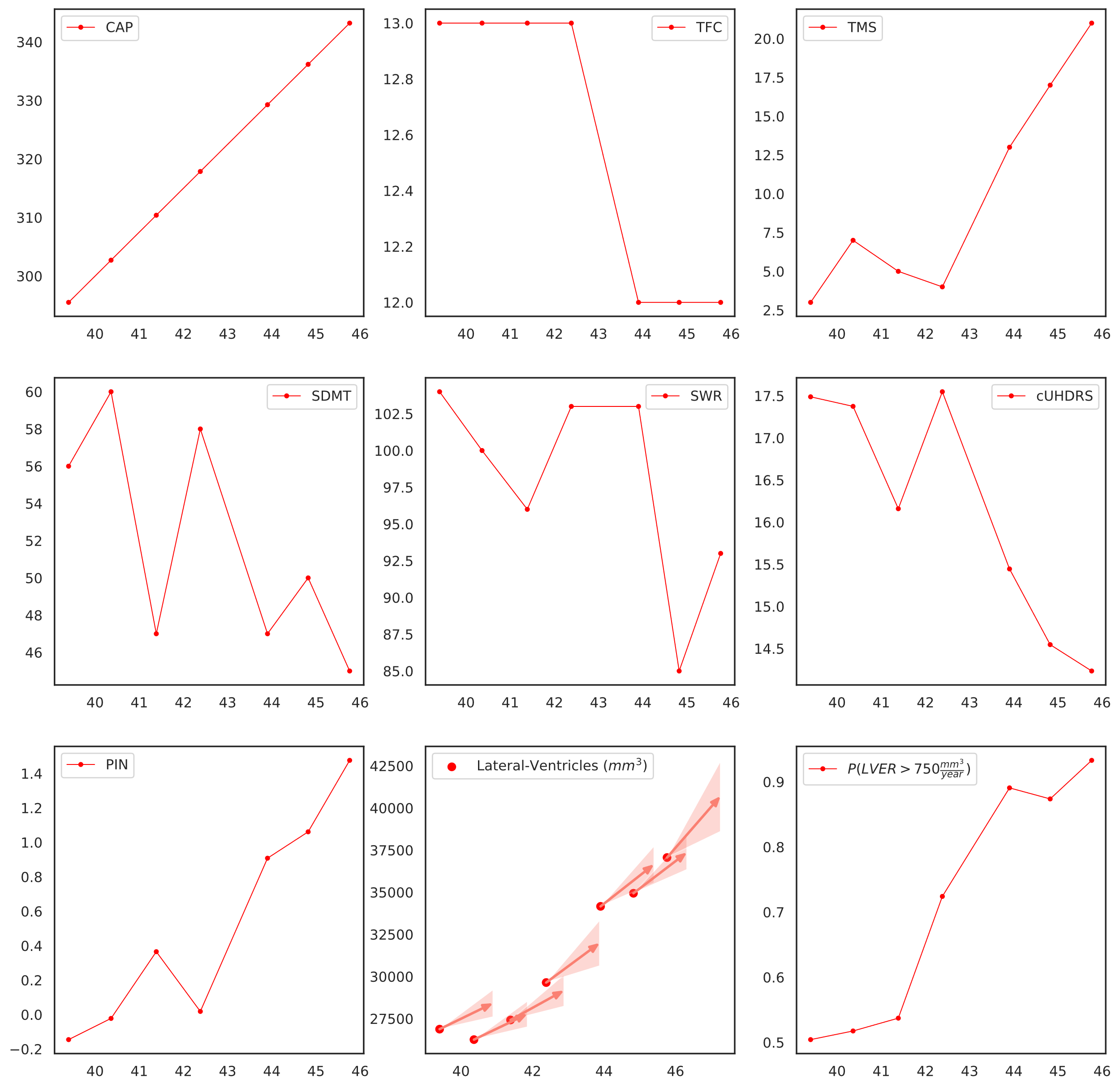


**Fig. 11 Conventional biomarkers of disease progression over 7 years vs prognostic and stratification models' outputs.** Considering the fluctuations in cognitive and motor assessments arising from practice effects, rater subjectivity, or variability, the PIN score is more susceptible to inconsistencies when compared to the greater robustness offered by the probabilistic approach calculated as P(LVER>750). It is a measure of how certain the stratification model is about the threshold of 750mm^3^/year being surpassed. In this example, P(LVER>750) is higher than 0.5 for all time points and consistently grows with time.

# Case study: Patient-specific model explanations

Fig. 12 shows the explanations of the stratification model when fed with the features at time point t_1_. The probability of fast progression given by $f(X)$ is 0.547, whereas the probability of moderate progression equals $1-f(X)=0.453$. This suggests that the forthcoming lateral ventricular enlargement is more likely to exceed the threshold of 750 mm^3^/year. SHAP values add up to $f(X)$ and each feature's contribution either raises or lowers the value of $f(X)$ from its expected value $E[f(X)]$ computed from the training data.

Fig. 13 shows the same plot for features at t_4_, highlighting why $f(x)$ has significantly increased due to contributions from the thalamus, accumbens, caudate, and other imaging features.


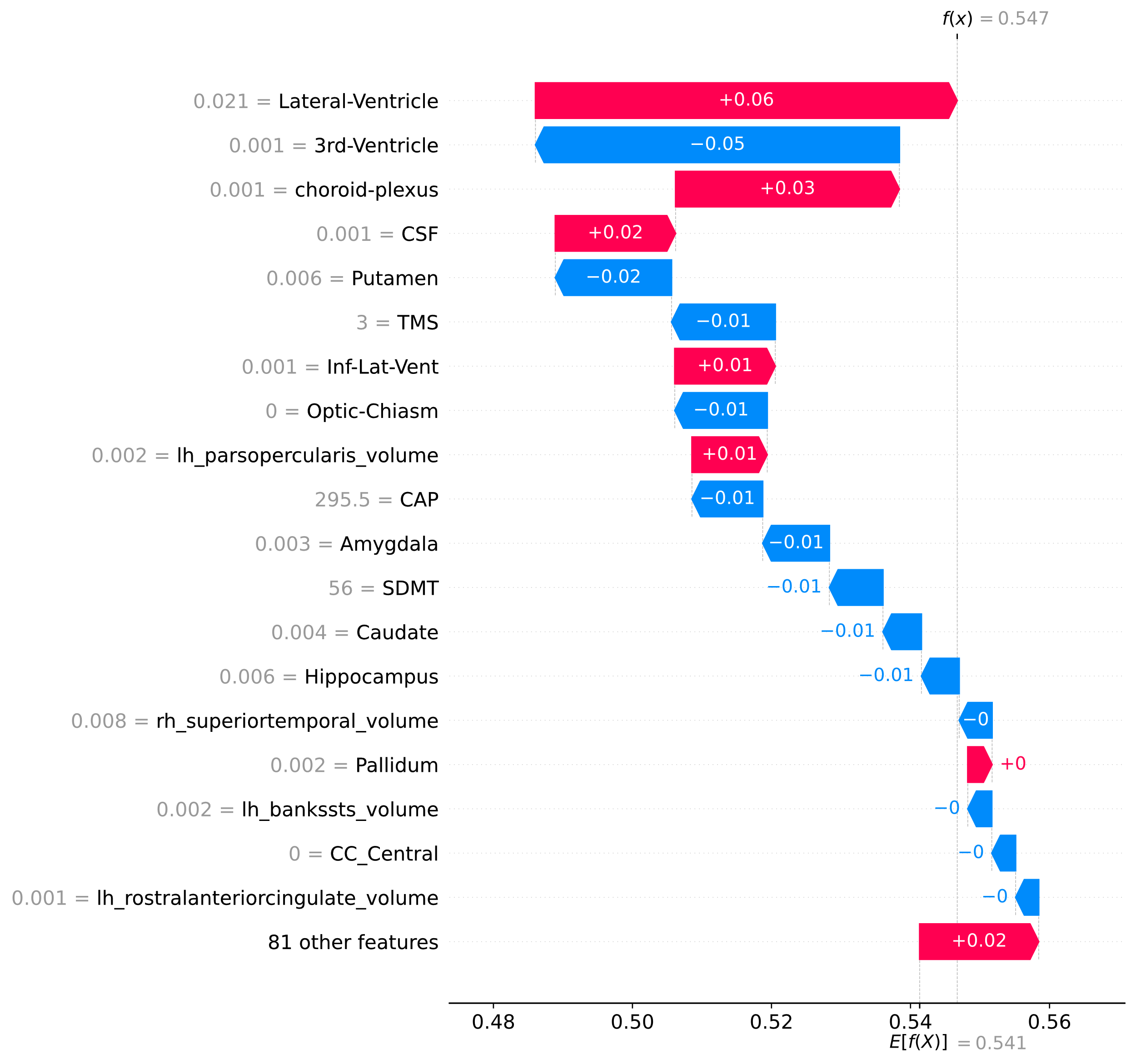


**Fig. 12 SHAP waterfall plot for time point t_1_.** Despite the relatively low and high values of TMS and SDMT respectively (in blue), the contributions from the lateral ventricles and choroid plexus (in red) have shifted the probability of fast progression.


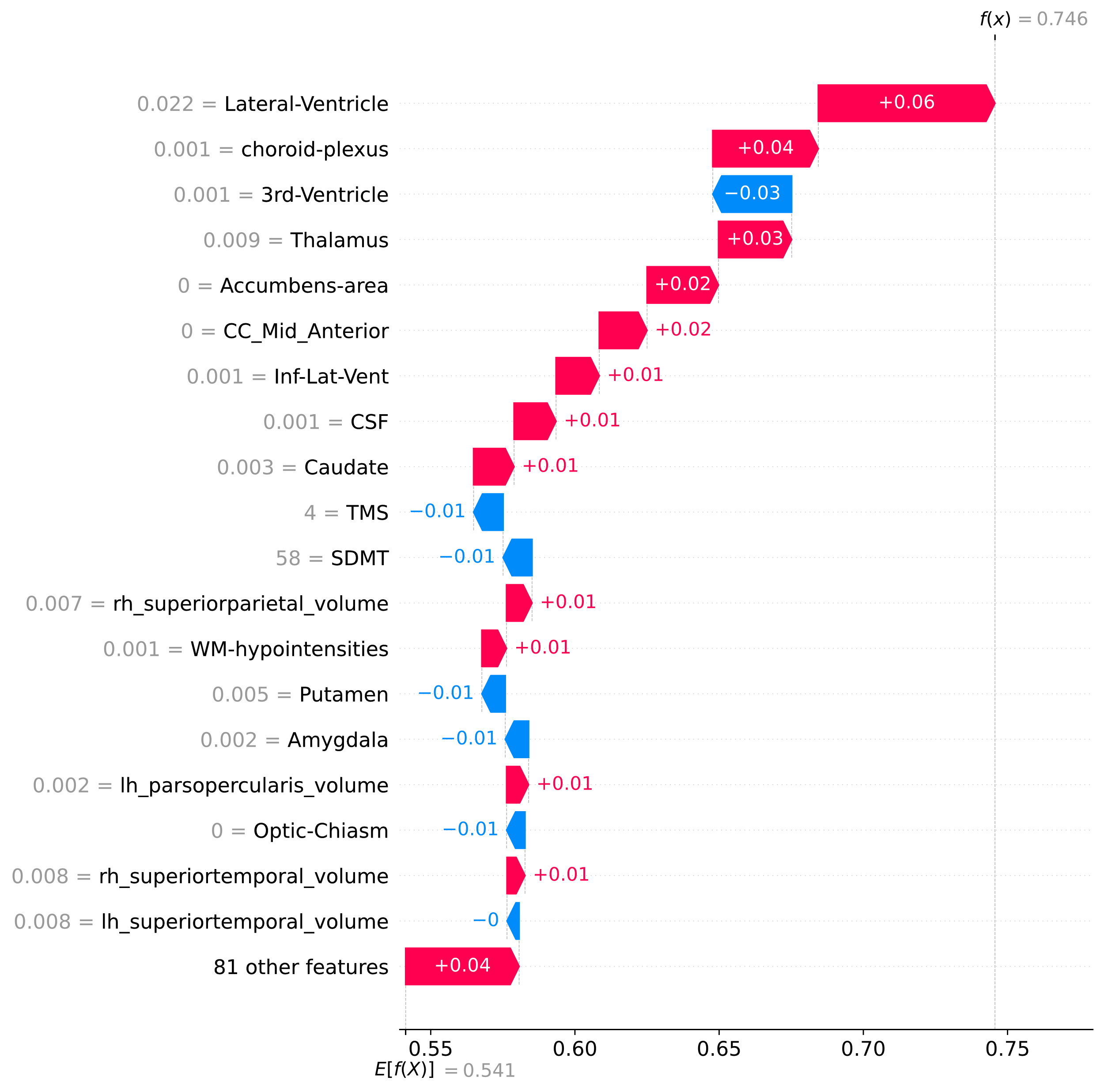


**Fig. 13 SHAP waterfall plot for time point t_4_.** Although TMS and SDMT have not drastically changed after three years (still in blue), the contributions coming from imaging derived features have significantly increased *f(X) =* ***P(****LVER>750 mm^3^/year****)***, highlighting the efficacy of the proposed probabilistic metric as a marker of disease progression and the applicability of the models for prognosis and stratification.

# Associations between striatal degeneration and ventricular enlargement

As Figure 3 of the manuscript show, smaller volumes of the baseline striatal structures correspond to faster LVER. For more clarification, here we provide cross-sectional and longitudinal analyses.

In our cross-sectional analyses, we investigated the associations between baseline striatum volume and the forthcoming lateral ventricular enlargement (LVER). As shown below, the volumes of striatal structures are strongly associated with LVER, with more atrophied striatum corresponding to faster forthcoming ventricular expansion:


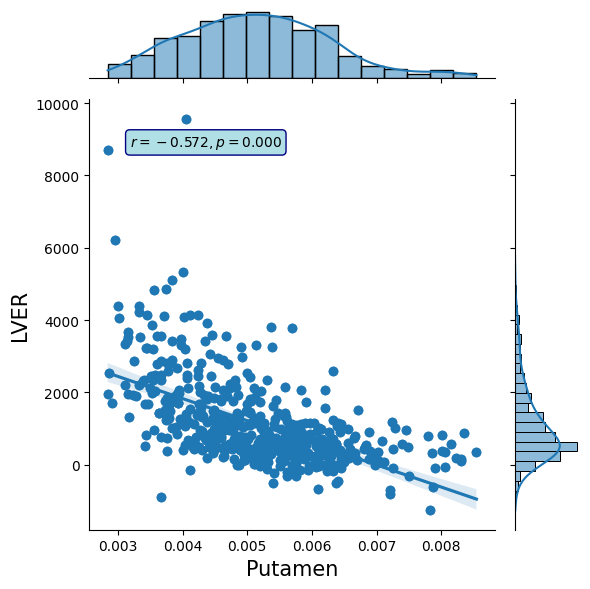

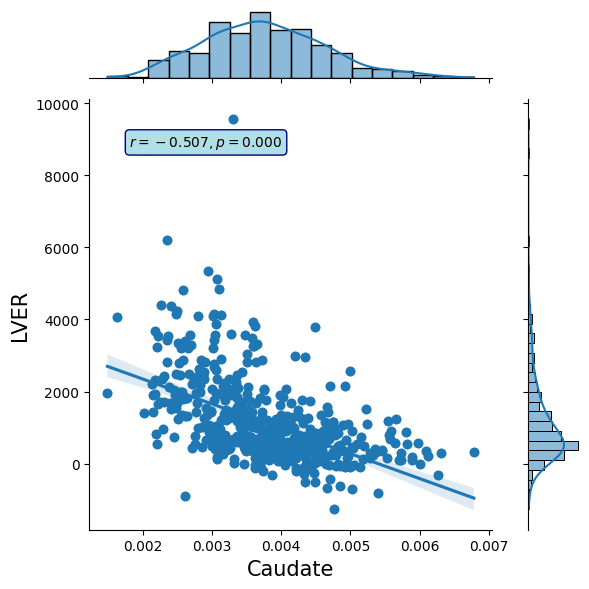


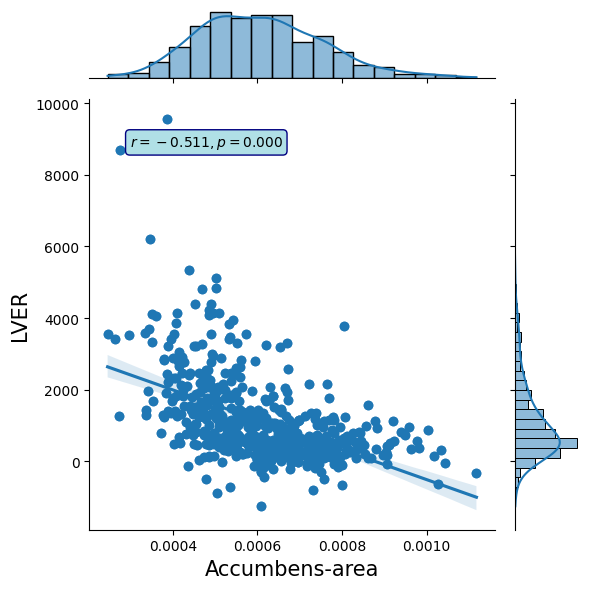

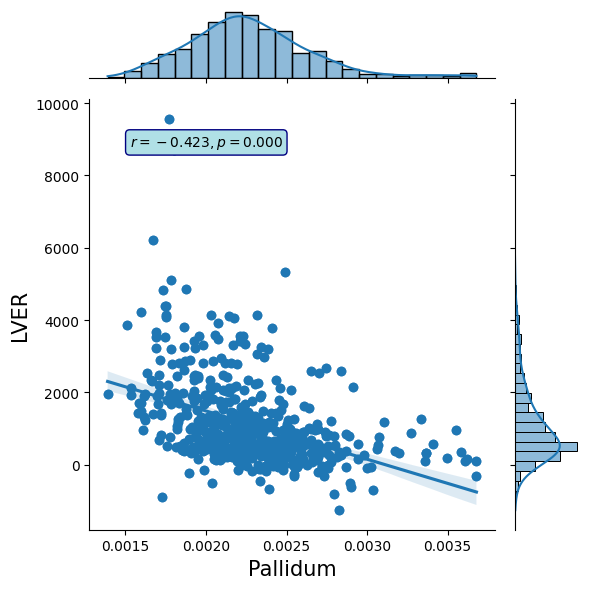
Cross-sectional associations between striatum and LVER

All grey matter structures are affected at some point in the Huntington’s disease course.^11,12^ The atrophies of these structures are synergistically reflected in ventricular expansion. Hobbs et al. reported that the atrophy of caudate and putamen in HD follow linear patterns, whereas the enlargement of the ventricles accelerates with disease duration, following a quadratic form.

In other words, a faster LVER does not necessarily imply faster atrophy of the structures in the striatum. Consequently, the correlation between putamen atrophy rate and LVER is weaker (-0.241 versus -0.572).


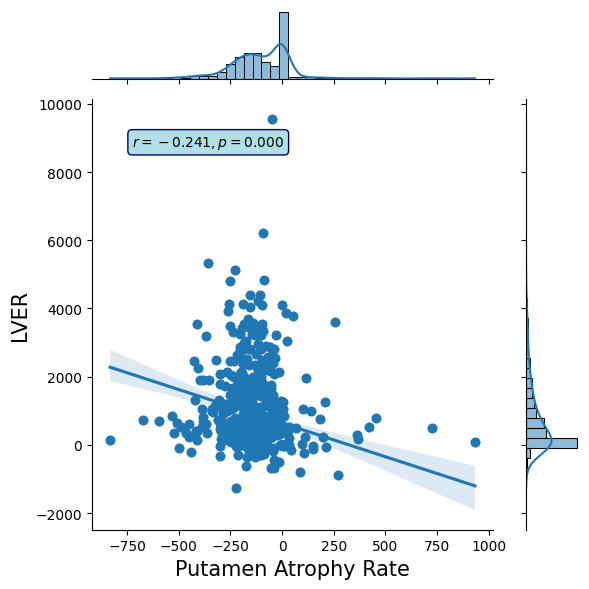

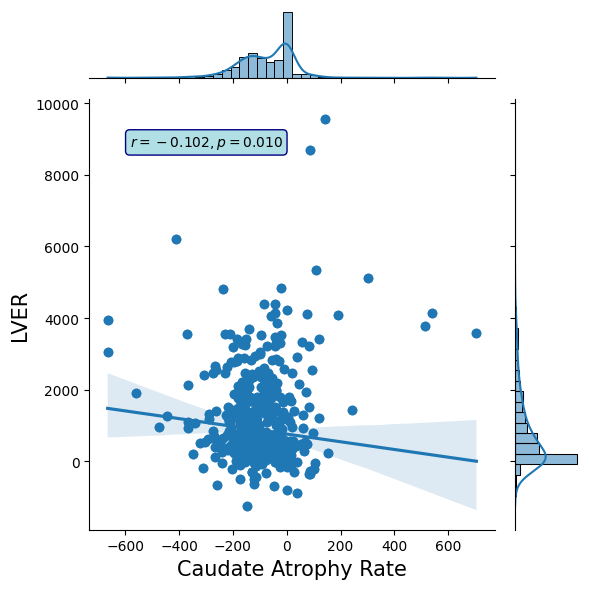


Longitudinal associations between caudate and putamen rate of atrophy and LVER

# The significance of quadratic-like pattern of lateral ventricular enlargement

Included below are the distribution of the volumes of the left caudate and the left lateral ventricle in 68,381 images from the UK biobank, as well as their coefficients of variation, $CV=\frac{\sigma}{\mu}$. As can be seen, the lateral ventricle has a skewed distribution with a much higher CV.

This is due to the fact that the enlargement of the ventricular volume generally follows a quadratic-like pattern throughout life as reported by Bethlehem et al. and Mofrad et al.^13,14^

This phenomenon is similarly evident in the pooled dataset consisting of 2,712 images of HD individuals. As pointed out in a study by Hobbs et al., the caudate (and putamen) atrophy in HD follows a linear pattern, whereas the enlargement of the ventricles accelerates with disease duration, following a quadratic form.^15^ We took advantage of this fact and predicted LVER by training random forest models using various relevant features as inputs. Quite intuitively, LVER also exhibit a skewed distribution as compared to ICV-normalized volume of caudate.

| 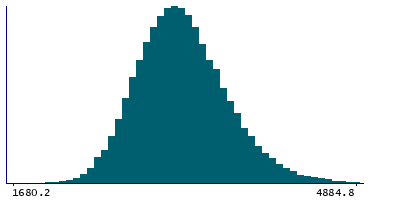 | 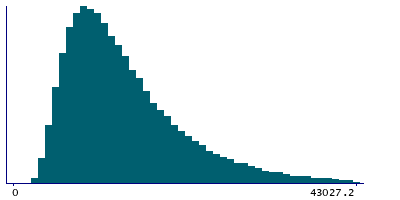 |
| --- | --- |
| **Left-Caudate (UKB)** | **Left-Lateral Ventricle (UKB)** |
| $CV=\frac{433.56}{3264.15}=0.133$ | $CV=\frac{8086.5}{14287.7}=0.566$ |
| 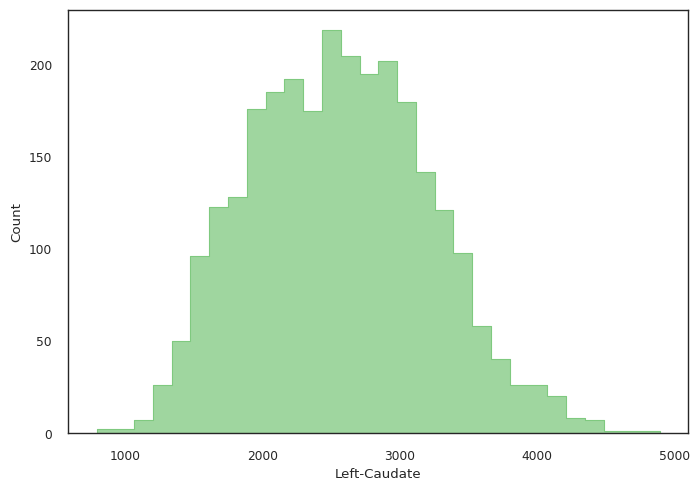 | 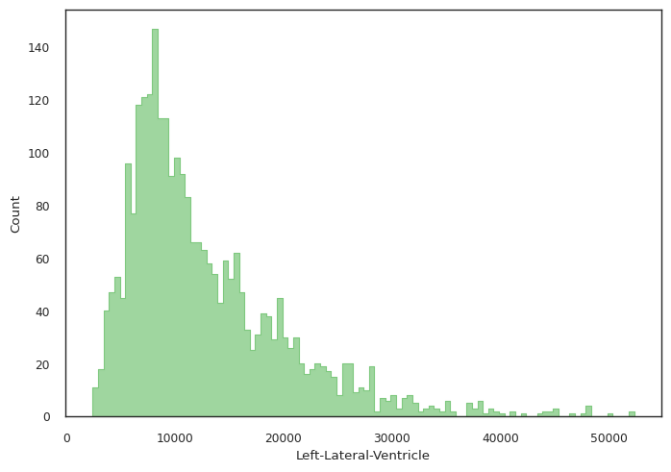 |
| **Left-Caudate (HD)** | **Left-Lateral Ventricle (HD)** |
| $CV=\frac{647.6}{2569.6}=0.252$ | $CV=\frac{7479.04}{12851.23}=0.582$ |
| 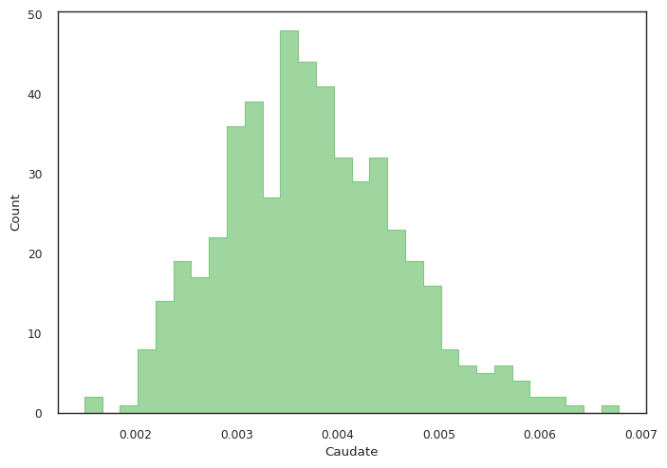 | 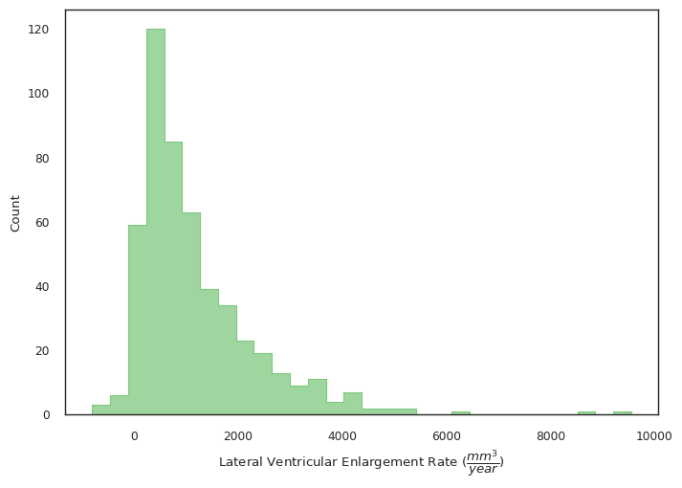 |
| **Caudate (HD)** | **LVER (HD)** |
| $CV=0.23$ | $CV=\frac{1184.6}{1179.8}=1.004$ |

Compare the following two figures to assess the variability in caudate volume versus the variability in LVER. The x-axes for the two figures represent the PIN score. For an arbitrary PIN score, the variability in LVER is much higher than the variability in caudate volume. In other words, compared to the caudate volume, LVER spans a significantly wider range of values, especially in HD-ISS stages 1 and 2. Therefore, LVER captures variations among individuals hence it is more sensitive compared to caudate volume. This is also quantitatively shown is Figure 5 in the manuscript, quantitively measured using kurtosis and entropy.


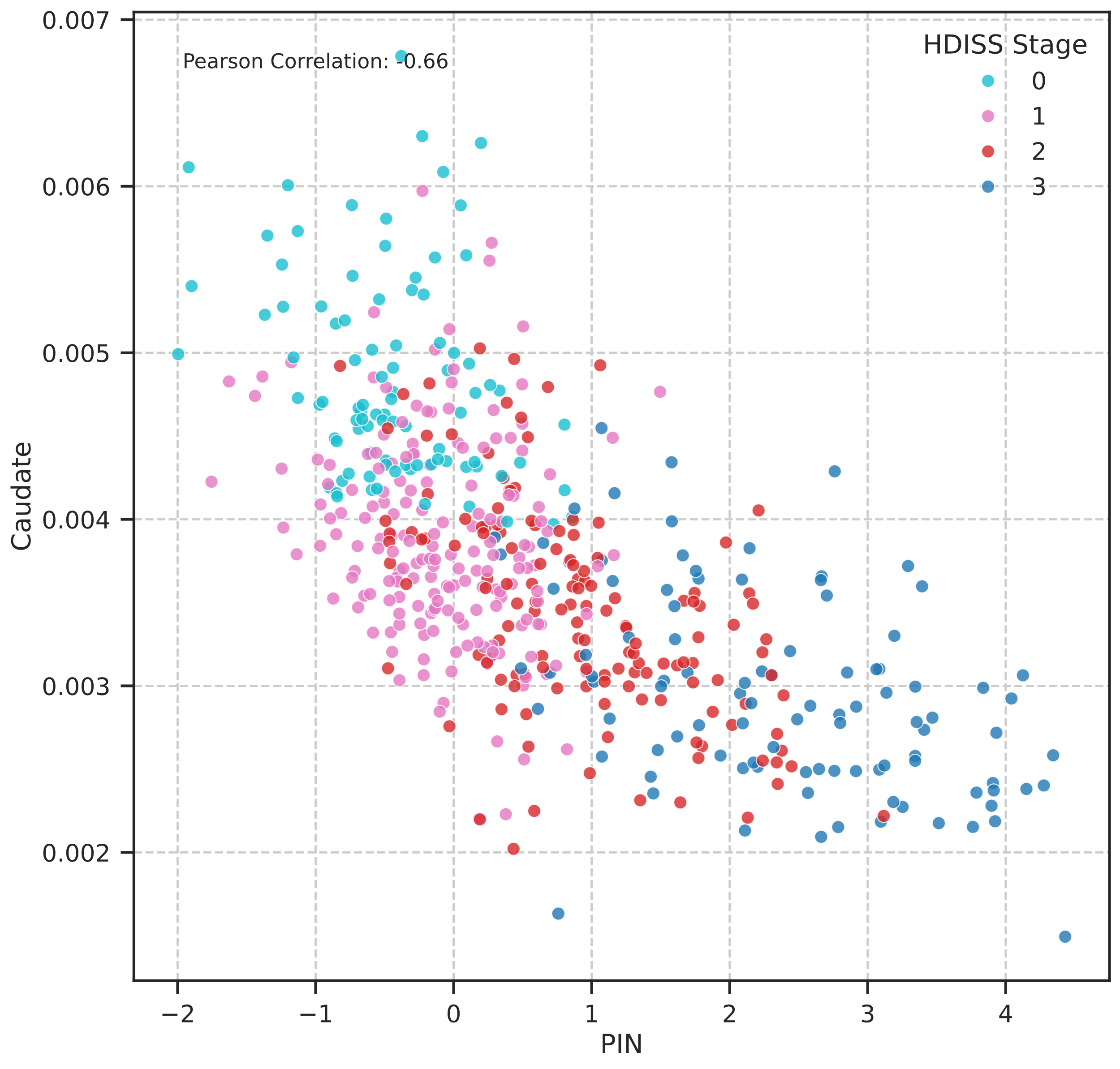

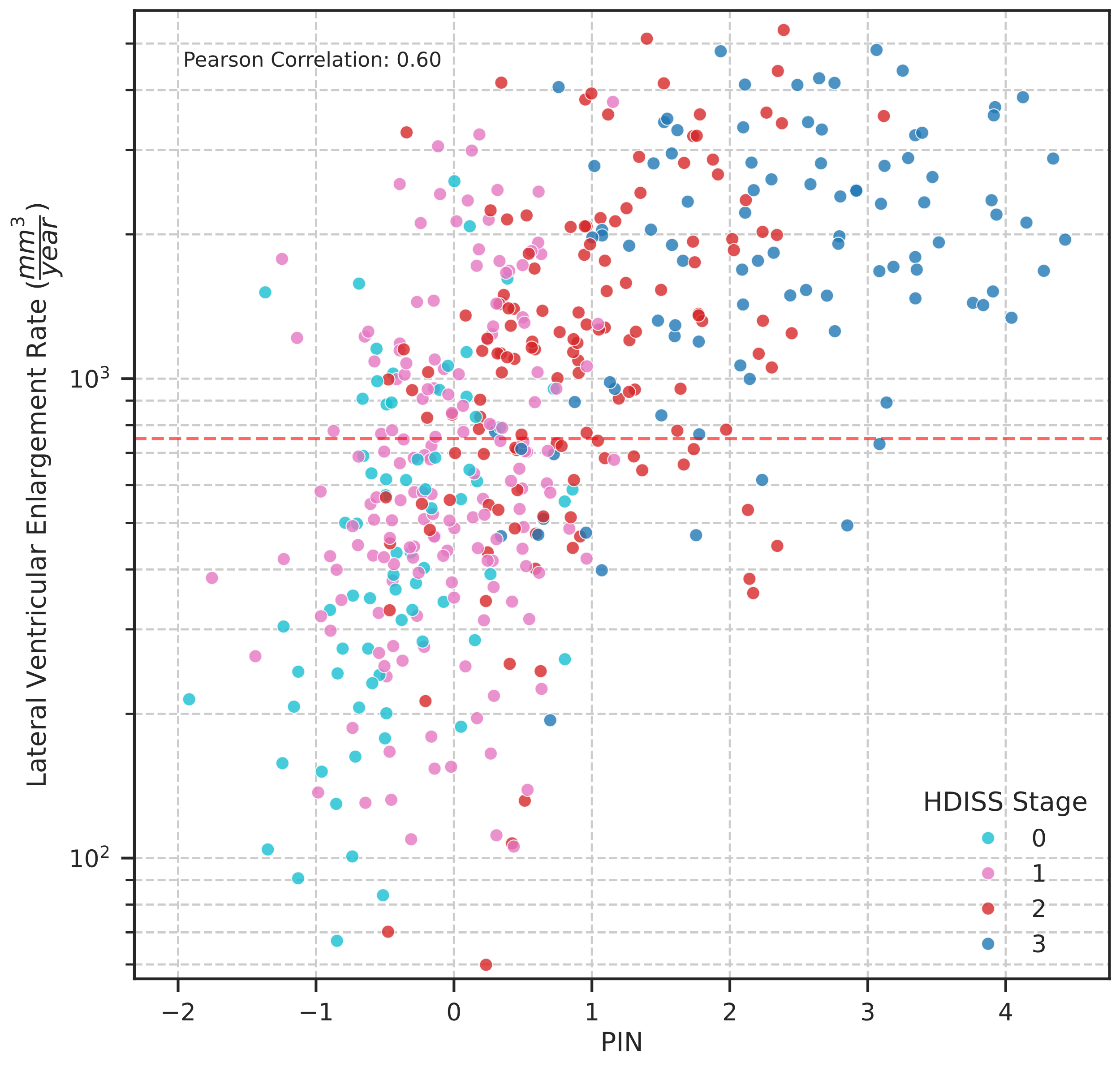


The variability of caudate volume versus LVER.

# Calculation of slopes using ordinary least squares

We acknowledged the fundamental practical necessity that, to be used in clinical trial, our proposed prognostic and stratification models should rely on measurements acquired in a single time-point, and not longitudinally. Leveraging our longitudinal dataset, the anticipated LVER was used as the target (dependent variable) to train our machine learning model, based on random forests. The example below shows how slope was calculated for an individual participating in TRACK-HD for 3.5 years.


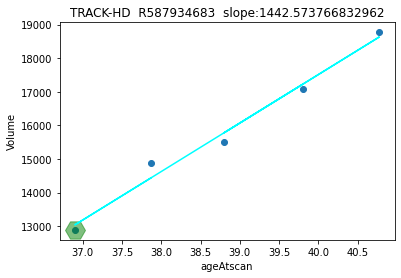


The decision to separate data for individuals with study engagements larger than 4 years, was made 1) to make LVER calculations more consistent, 2) to avoid underestimation or overestimation, and 3) based on the notion that a phase of a Huntington’s disease clinical trial is typically conducted within a time frame of 1~2 years.^16^ The duration of engagement in the three studies varied among individuals (Table 1 in the manuscript). Figures below show the number of individuals based on their length of engagement in IMAGE, TRACK and PREDICT.


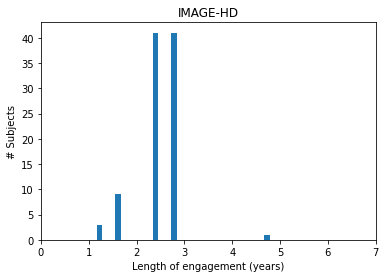

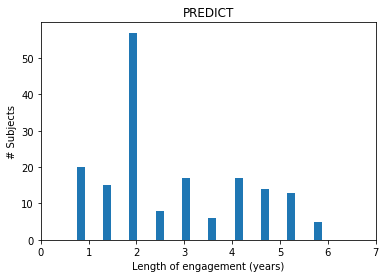


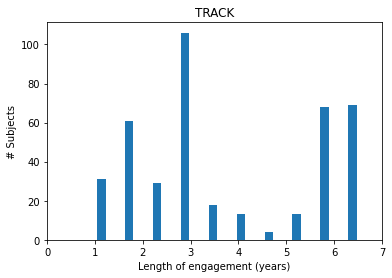


We repeated our model training and evaluation for the case where all time points were included without separation into two segments. So, instead of having 537 input-output pairs, we obtained 451 pairs, equal to the number of individuals in our dataset. However, cross-validated accuracy and unseen data accuracy did not change significantly.

| 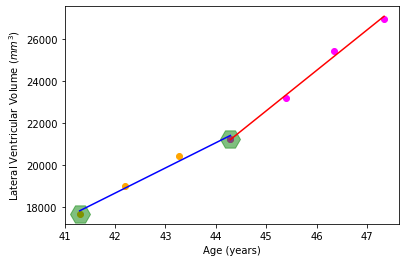 | 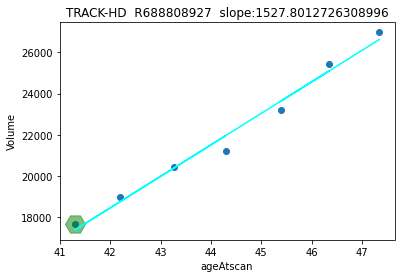 |
| --- | --- |
| a) Assignment of two baseline-slope pairs | b) time points included **without** separation into segments |

**References**

1. Wei K, Tran T, Chu K, et al. White matter hypointensities and hyperintensities have equivalent correlations with age and CSF β‐amyloid in the nondemented elderly. *Brain Behav*. 2019;9(12):e01457.

2. Althubaity N, Schubert J, Martins D, et al. Choroid plexus enlargement is associated with neuroinflammation and reduction of blood brain barrier permeability in depression. *NeuroImage Clin*. 2022;33:102926. doi:10.1016/j.nicl.2021.102926

3. Zhou G, Hotta J, Lehtinen MK, Forss N, Hari R. Enlargement of choroid plexus in complex regional pain syndrome. *Sci Rep*. 2015;5:14329. doi:10.1038/srep14329

4. Zeng J, Zhang T, Tang B, et al. Choroid plexus volume enlargement in first-episode antipsychotic-naïve schizophrenia. *Schizophr (Heidelberg, Ger*. 2024;10(1):1. doi:10.1038/s41537-023-00424-2

5. Dai T, Lou J, Kong D, et al. Choroid plexus enlargement in amyotrophic lateral sclerosis patients and its correlation with clinical disability and blood-CSF barrier permeability. *Fluids Barriers CNS*. 2024;21(1):36. doi:10.1186/s12987-024-00536-6

6. Andravizou A, Stavropoulou De Lorenzo S, Kesidou E, et al. The Time Trajectory of Choroid Plexus Enlargement in Multiple Sclerosis. *Healthcare*. 2024;12(7). doi:10.3390/healthcare12070768

7. Umemura Y, Watanabe K, Kasai S, et al. Choroid plexus enlargement in mild cognitive impairment on MRI: a large cohort study. *Eur Radiol*. Published online January 2024. doi:10.1007/s00330-023-10572-9

8. Efron B, Hastie T, Johnstone I, Tibshirani R. Least angle regression. Published online 2004.

9. Sweidan W, Bao F, Bozorgzad N, George E. White and Gray Matter Abnormalities in Manifest Huntington’s Disease: Cross‐Sectional and Longitudinal Analysis. *J Neuroimaging*. 2020;30(3):351-358. doi:10.1111/jon.12699

10. Coppen EM, Grond J van der, Hafkemeijer A, Barkey Wolf JJH, Roos RAC. Structural and functional changes of the visual cortex in early Huntington’s disease. *Hum Brain Mapp*. 2018;39(12):4776-4786. doi:10.1002/hbm.24322

11. Van Den Bogaard SJA, Dumas EM, Acharya TP, et al. Early atrophy of pallidum and accumbens nucleus in Huntington’s disease. *J Neurol*. 2011;258(3):412-420. doi:10.1007/s00415-010-5768-0

12. Wijeratne PA, Young AL, Oxtoby NP, et al. An image-based model of brain volume biomarker changes in Huntington’s disease. *Ann Clin Transl Neurol*. 2018;5(5):570-582. doi:10.1002/acn3.558

13. Bethlehem RAI, Seidlitz J, White SR, et al. Brain charts for the human lifespan. *Nature*. 2022;604(7906):525-533. doi:10.1038/s41586-022-04554-y

14. Mofrad SA, Lundervold A, Lundervold AS. A predictive framework based on brain volume trajectories enabling early detection of Alzheimer’s disease. *Comput Med Imaging Graph*. 2021;90(February):101910. doi:10.1016/j.compmedimag.2021.101910

15. Hobbs NZ, Barnes J, Frost C, et al. Onset and progression of pathologic atrophy in Huntington disease: A longitudinal MR imaging study. *Am J Neuroradiol*. 2010;31(6):1036-1041. doi:10.3174/ajnr.A2018

16. Kinnunen KM, Schwarz AJ, Turner EC, et al. Volumetric MRI-Based Biomarkers in Huntington’s Disease: An Evidentiary Review. *Front Neurol*. 2021;12(September). doi:10.3389/fneur.2021.712555
